# New Isocoumarin Analogues from the Marine-Derived Fungus *Paraphoma* sp. CUGBMF180003

**DOI:** 10.3390/md19060313

**Published:** 2021-05-28

**Authors:** Xiuli Xu, Jiangpeng Li, Kai Zhang, Shangzhu Wei, Rui Lin, Steven W. Polyak, Na Yang, Fuhang Song

**Affiliations:** 1 School of Ocean Sciences, China University of Geosciences, Beijing 100083, China; xuxl@cugb.edu.cn (X.X.); lijiangpeng@cugb.edu.cn (J.L.); weishangzhu@cugb.edu.cn (S.W.); linrui@cugb.edu.cn (R.L.); 2 School of Light Industry, Beijing Technology and Business University, Beijing 100048, China; zhangkai2030302071@st.btbu.edu.cn; 3 UniSA Clinical and Health Sciences, University of South Australia, Adelaide 5005, Australia; Steven.Polyak@unisa.edu.au; 4 CAS Key Laboratory of Experimental Marine Biology, Institute of Oceanology, Chinese Academy of Sciences, Qingdao 266071, China; 5 Laboratory for Marine Biology and Biotechnology, Qingdao National Laboratory for Marine Science and Technology, Qingdao 266071, China

**Keywords:** marine-derived fungus, *Paraphoma* sp., natural products, isocoumarin derivatives, antibacterial activity

## Abstract

Nine new secondary metabolites, including six isocoumarin analogues, 7-hydroxyoospolactone (**1**), 7-methoxyoospolactone (**2**), 7-methoxy-9-hydroxyoospolactone (**3**), 10-acetoxy-9-hydroxyoospolactone (**4**), 6-dehydroxysescandelin (**5**), parapholactone (**6**), and three compounds with a rare skeleton of isocoumarin coupled with phenylethylamine, namely paraphamide A (**12**), paraphamide B (**13**), and paraphamide C (**14**), together with five known compounds, oospolactone (**7**), 8-*O*-methyloospolactone (**8**), 10-hydroxyoospolactone (**9**), 9,10-dihydroxyoospolactone (**10**), and oospoglycol (**11**), were isolated and identified from the marine-derived fungus *Paraphoma* sp. CUGBMF180003. Their chemical structures were determined using spectroscopic data, including HRESIMS and 1D and 2D NMR techniques. Furthermore, the stereogenic carbons in **5** and **14** were determined by comparing the experimental and calculated electronic circular dichroism (ECD) spectra. The carbon skeleton of **12**–**14** was identified as the first example of isocoumarin coupled with phenylethylamine derivatives. All of these compounds were examined for antimicrobial activities against *Candida albicans* and *Staphylococcus aureus.* Both **1** and **6** showed antibacterial activity against *S. aureus* with MIC values of 12.5 μg/mL.

## 1. Introduction

The *Paraphoma* fungi are commonly isolated from soil samples or plants and recognized as soil-borne pathogens [1,2]. Strains belonging to this genus have been proven to degrade plastic films such as poly(butylene succinate-co-butylene adipate) and poly(butylene succinate) [3,4]. Some secondary metabolites, such as polyketides [5], phenol, latam [6], and isochromenones [7] have been identified in fungi of this genus. Our present chemical investigation on the marine-derived fungus *Paraphoma* sp. strain CUGBMF180003, isolated from a mud sample collected from Shenzhen, China, led to the identification of nine new secondary metabolites, including six isocoumarin analogues, namely, 7-hydroxyoospolactone (**1**), 7-methoxyoospolactone (**2**), 7-methoxy-9-hydroxyoospolactone (**3**), 10-acetoxy-9-hydroxyoospolactone (**4**), 6-dehydroxysescandelin (**5**), parapholactone (**6**), three compounds with a rare skeleton of isocoumarin coupled with phenylethylamine, namely, paraphamide A (**12**), paraphamide B (**13**), and paraphamide C (**14**), as well as five known compounds, oospolactone (**7**) [8], 8-*O*-methoxyoospolactone (**8**) [9], 10-hydroxyoospolactone (**9**) [10], 9,10-dihydroxyoospolactone (**10**) [10], and oospoglycol (**11**) [11]. Herein, we report the isolation, structure elucidation, and biological activities of these compounds. 

## 2. Results

### 2.1. Structure Elucidation

**Figure 1:**
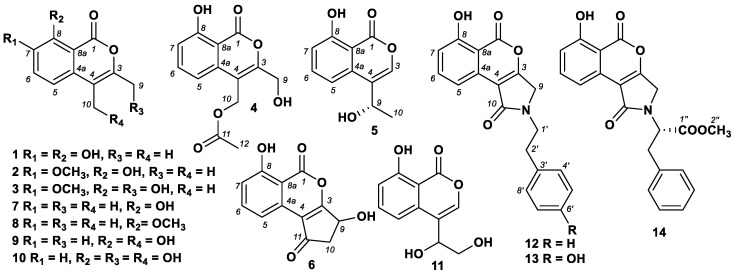
Chemical structures of **1**–**14**.

Compound **1** was isolated as a light yellow powder. The molecular formula of **1** was determined to be C_11_H_10_O_4_ based on the HRESIMS spectrum (*m/z* [M + H]^+^ 207.0652, calcd. for C_11_H_11_O_4_, 207.0652), accounting for seven degrees of unsaturation (Figure S1). The ^1^H NMR data of **1** (Table 1, Figure S2) demonstrated two aromatic proton signals [(*δ*_H_ 6.93, d, *J* = 8.5 Hz, H-5), (*δ*_H_ 7.31, 1H, d, *J* = 8.5 Hz, H-6)], two methyl singlets at *δ*_H_ 2.24 (3H, s, H-9) and 2.06 (3H, s, H-10), and two phenolic hydroxyl groups at *δ*_H_ 9.69 (1H, brs, 7-OH) and 11.12 (1H, brs, 8-OH). The ^13^C and HSQC spectra of **1** (Figures S3 and S4) indicated 11 carbon signals (Table 1) which were categorized as one carboxyl at *δ*_C_ 166.2 (C-1), three oxygenated sp^2^ carbons at *δ*_C_ 146.7 (C-3), 143.8 (C-7), and 148.6 (C-8), two nonprotonated sp^2^ carbons at *δ*_C_ 129.6 (C-4a) and 105.9 (C-8a), and two protonated sp^2^ carbons at *δ*_C_ 113.3 (C-5) and 124.2 (C-6). Detailed analysis of the 2D NMR data (Figures S4–S6) revealed the existence of an isocoumarin skeleton for **1**. The HMBC correlations from H-5 to C-7 and C-8a, from H-6 to C-4a, C-7 and C-8 revealed the *o*-dihydroxyl substituted benzene moiety. The long-range HMBC correlation from H-5 to C-1 indicated the presence of a carboxyl substituent on C-8a. Furthermore, the HMBC correlations from H-9 and H-10 to C-3 and C-4 indicated that the methyl groups were on C-3 and C-4. The connection from C-4 to C-4a was evidenced by the HMBC correlation from H-10 to C-4a. With the downfield chemical shifts of C-1 and C-3, it was revealed that C-1 and C-3 were connected by an oxygen atom. Therefore, the structure of **1** was assigned as shown in Figure 1 and named 7-hydroxyoospolactone.

Compound **2** was isolated as a light yellow powder. The molecular formula of **2** was determined to be C_12_H_12_O_4_ based on the HRESIMS spectrum (*m/z* [M + H]^+^ 221.0811, calcd. for C_12_H_13_O_4_, 221.0808), accounting for seven degrees of unsaturation (Figure S7). The ^1^H NMR data of **2** (Table 1, Figure S8) displayed similar signals to those of **1**. Comparison of the NMR data of the two compounds revealed the presence of one methoxyl signal at *δ*_H_ 3.85, s (*δ*_C_ 56.1) in **2**. The HMBC spectrum (Figure S12) exhibited correlations from H-5 and -OMe to C-7, indicating that the methoxyl group was on C-7. Therefore, the structure of **2** was elucidated as shown in Figure 1 and named 7-methoxyoospolactone.

**Figure 2:**
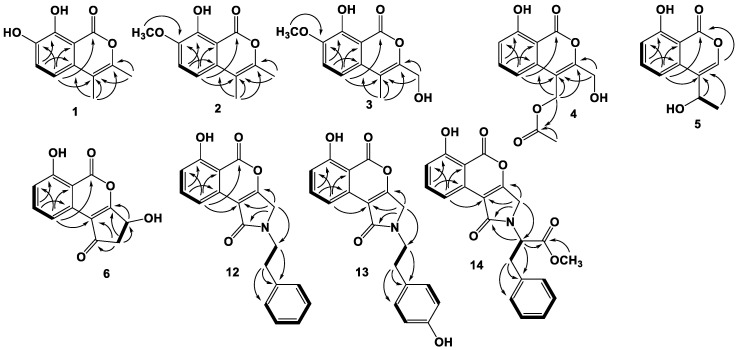
Key COSY (bold lines) and HMBC (arrows) correlations in **1**–**6** and **12**–**14**.

Compound **3** was isolated as a light yellow powder. The molecular formula of **3** was determined to be C_12_H_12_O_5_ based on the HRESIMS spectrum (*m/z* [M + H]^+^ 237.0758, calcd. for C_12_H_13_O_5_, 237.0757), accounting for seven degrees of unsaturation (Figure S13). The ^1^H NMR data of **3** (Table 1, Figure S14) displayed similar signals to those of **2**, except for the presence of the signal of hydroxymethyl protons *δ*_H_ 4.36 (2H, s, H-9; *δ*_C_ 57.6 C-9). Detailed analysis of ^13^C and 2D NMR data (Figures S15–S17) revealed the structure of **3**. The HMBC spectrum (Figure 2, Figure S17) exhibited correlations from H-9 to C-3 and C-4, and from H-10 to C-3, C-4, and C-4a indicating that the hydroxymethyl group was on C-3. Therefore, the structure of **3** was elucidated as shown in Figure 1 and named 7-methoxy-9-hydroxyoospolactone.

Compound **4** was isolated as a light yellow powder. The molecular formula of **4** was determined to be C_13_H_12_O_6_ based on the HRESIMS spectrum (*m/z* [M + H]^+^ 265.0708, calcd. for C_13_H_13_O_6_, 265.0707), accounting for eight degrees of unsaturation (Figure S18). The ^1^H NMR data of **4** (Table 2, Figure S19) displayed signals of three aromatic protons at *δ*_H_ 7.33 (1H, d, *J =* 8.0 Hz, H-5), 7.70 (1H, dd, *J =* 8.0, 8.0 Hz, H-6), and 7.06 (1H, d, *J =* 8.0 Hz, H-7), two hydroxymethyl protons at *δ*_H_ 4.82 (2H, s, H-9), and two oxymethylene protons at *δ*_H_ 5.09 (2H, s, H-10), as well as one methyl singlet at *δ*_H_ 2.12 (3H, s, H-12). The ^13^C and HSQC spectra of **4** (Figures S20 and S21) showed 13 carbon signals, including those of the isocoumarin skeleton similar to those of oospolactone, and the two methyls in oospolactone were replaced by two hydroxymethyls at *δ*_C_ 57.1 (C-9) and 60.1 (C-10), as well as two signals for acetyl groups at *δ*_C_ 171.8 (C-11) and 21.0 (C-12). The presence of the hydroxymethyl group on C-3 was confirmed by the HMBC correlations (Figure S23) from H-9 to C-3 and C-4. Additionally, the HMBC correlations from H-10 to C-3, C-4, C-4a, and C-11 and from H-12 to C-11 revealed an acetoxy group on C-10. Thus, the structure of **4** was elucidated as shown in Figure 1 and named 10-acetoxy-9-hydroxyoospolactone.

**Figure 3:**
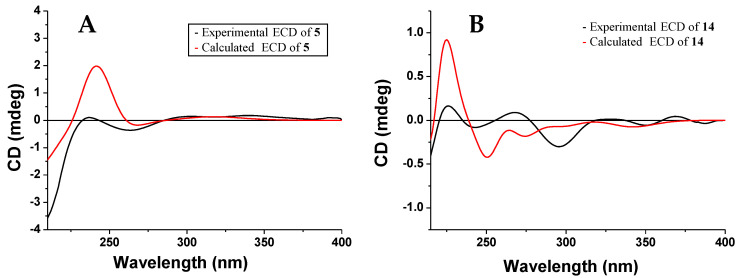
Calculated and experimental electronic circular dichroism (ECD) spectra of 5 (**A**) and 14 (**B**).

Compound **5** was isolated as a light yellow powder. The molecular formula of **5** was determined to be C_11_H_10_O_4_ based on the HRESIMS spectrum (*m/z* [M + H]^+^ 207.0648, calcd. for C_11_H_11_O_4_, 207.0652), accounting for seven degrees of unsaturation (Figure S24). The ^1^H data of **5** (Table 2, Figure S25) displayed three aromatic proton signals at *δ*_H_ 7.13 (1H, d, *J =* 8.0 Hz, H-5), 7.66 (1H, dd, *J =* 8.0, 8.0 Hz, H-6), and 7.02 (1H, d, *J =* 8.0 Hz, H-7), one olefinic proton signal at 7.37 (1H, s, H-3), one methyl doublet at *δ*_H_ 1.60 (3H, d, *J =* 6.5 Hz, H-10), and one oxygenated sp^3^ methine at *δ*_H_ 5.06 (1H, q, *J =* 6.5 Hz, H-9). The ^13^C and HSQC spectra (Figures S26 and S27) presented 11 carbon resonances, including one carboxyl at *δ*_C_ 166.4 (C-1), four protonated sp^2^ at *δ*_C_ 141.2 (C-3), 113.4 (C-5), 137.4 (C-6), 116.0 (C-7), four nonprotonated sp^2^ carbon signals at *δ*_C_ 122.1 (C-4), 135.7 (C-4a), 162.4 (oxygenated, C-8), and 106.8 (C-8a), as well as one signal for oxygenated methine sp^3^ at *δ*_C_ 65.0 (C-9) and one methyl carbon at *δ*_C_ 23.3 (C-10). Detailed analysis of the 2D NMR data (Figures S27–S29) revealed that **5** is an analogue of the isocoumarin oospolactone. However, the methyl group at C-3 was replaced by a proton, and another methyl group at C-4 was replaced by a 1-hydroxyethyl group. The proposed structure was confirmed by the HMBC correlations from H-3 to C-1, C-4a, C-9, and C-10, and from H-10 to C-4 and C-9. Thus, the planar structure of **5** was elucidated. The absolute configuration of C-9 was assessed by comparison of experimental and calculated ECD spectra (Figure 3A), confirming the *S* configuration of C-9, and the compound was named 6-dehydroxysescandelin.

Compound **6** was isolated as a light yellow powder. The molecular formula of **6** was determined to be C_12_H_8_O_5_ based on the HRESIMS spectrum (*m/z* [M + H]^+^ 233.0443, calcd. for C_12_H_9_O_5_, 233.0444), accounting for nine degrees of unsaturation (Figure S30). The ^1^H NMR data of **6** (Table 2, Figure S31) displayed signals of three aromatic protons at *δ*_H_ 7.50 (1H, dd, *J =* 7.5, 1.0 Hz, H-5), 7.69 (1H, dd, *J =* 8.5, 7.5 Hz, H-6), and 7.04 (1H, dd, *J =* 8.5, 1.0 Hz, H-7), two methylene sp^3^ protons at *δ*_H_ 3.06 (1H, dd, *J =* 18.0, 6.0 Hz, H-10a) and 2.76 (1H, dd, *J =* 18.0, 3.0 Hz, H-10b), and one oxygenated methine sp^3^ proton at *δ*_H_ 5.83 (1H, dd, *J =* 6.0, 3.0 Hz, H-9). The ^13^C NMR and HSQC spectra (Figures S32 and S33) revealed twelve carbon resonances including one ketone carbonyl at *δ*_C_ 196.4 (C-11), one carboxyl at *δ*_C_ 163.2 (C-1), three protonated sp^2^ at *δ*_C_ 118.9 (C-5), 137.5 (C-6), 119.0 (C-7), three nonprotonated sp^2^ at *δ*_C_ 127.3 (C-4), 133.9 (C-4a), and 107.8 (C-8a), two oxygenated sp^2^ at *δ*_C_ 151.1 (C-3) and 163.2 (C-8), and one methylene sp^3^ at *δ*_C_ 39.9 (C-10) and one oxymethine sp^3^ at *δ*_C_ 75.3 (C-9). Detailed analysis of the 2D NMR data of **6** (Figures S33–S35) revealed an isocoumarin analogue. HMBC correlations from H-10 to C-3, C-4, and C-11, and from H-9 to C-3 and C-4 suggested that the 4-hydroxycyclopentenone ring was fused with the chromone ring through C-3 and C-4. Compound **6** showed weak optical rotation value of +0.91 (*c* 0.11, MeOH) and did not display ECD absorptions; therefore, **6** was elucidated as a racemic mixture. Therefore, the structure of **6** was defined as shown in Figure 1 and named parapholactone. 

Compound **12** was isolated as a light yellow powder. The molecular formula of **12** was determined to be C_19_H_15_NO_4_ based on the HRESIMS spectrum (*m/z* [M + H]^+^ 322.1069, calcd. for C_19_H_16_NO_4_, 322.1074), accounting for thirteen degrees of unsaturation (Figure S36). The ^1^H and ^13^C NMR data of **12** (Table 3, Figures S37 and S38) displayed proton and carbon signals of isocoumarin moiety similar to those of oospolactone, as well as resonances for one phenylethyl group [*δ*_H_ 3.73, t, *J* = 7.5 Hz, H-1′, *δ*_C_ 43.7 C-1′; *δ*_H_ 2.93, t, *J* = 7.5 Hz, H-2′, *δ*_C_ 33.8 C-2′; *δ*_H_ 7.27, d, *J* = 7.0 Hz, H-4′/8′, *δ*_C_ 128.6 C-4′/8′; *δ*_H_ 7.30, dd, *J* = 7.0, 7.0 Hz, H-5′/7′, *δ*_C_ 128.4 C-5′/7′; *δ*_H_ 7.21, t, *J* = 7.0 Hz, H-6′, *δ*_C_ 126.3 C-6′], one methylene at *δ*_H_ 4.48 (s, H-9), *δ*_C_ 45.6 (C-9) and one conjugated amide carbonyl at *δ*_C_ 160.9 (C-10). Detailed analysis of 2D NMR data (Figures S39–S41) confirmed the connections of the phenylethyl moiety to the isocoumarin core. The molecular formula (C_19_H_15_NO_4_) of **12** and downfield shift of C-9 (*δ*_C_ 45.6), combined with the HMBC correlations from H-9 to C-3, C-4, C-10, and C-1′, and from H-1′ to C-9 and C-10 confirmed the connection of C-1′, C-9, and C-10 to N, forming a cyclopentenamide ring. Therefore, the structure of **12** was elucidated as shown in Figure 1 and named paraphamide A.

Compound **13** was isolated as a light yellow powder. The molecular formula of **13** was determined to be C_19_H_15_NO_5_ based on the HRESIMS spectrum (*m/z* [M + H]^+^ 338.1022, calcd. for C_19_H_16_NO_5_, 338.1023), accounting for thirteen degrees of unsaturation (Figure S42). The ^1^H and ^13^C NMR spectra of **13** (Table 3, Figures S43 and S44) resembled those of **12**. Detailed analysis of the ^1^H and ^13^C NMR data (Figures S43 and S44) revealed that H-6′ of **12** was replaced by a hydroxyl group in **13**. The structure of **13** was confirmed by the downfield shift of C-6′ (*δ*_C_ 155.8) and molecular formula. Thus, the structure of **13** was elucidated as shown in Figure 1 and named paraphamide B.

Compound **14** was isolated as a light yellow powder. The molecular formula of **14** was determined to be C_21_H_17_NO_6_ based on the HRESIMS spectrum (*m/z* [M + H]^+^ 380.1130, calcd. for C_21_H_18_NO_6_, 380.1129), accounting for fourteen degrees of unsaturation (Figure S48). The ^1^H and ^13^C NMR spectra of **14** (Table 3, Figures S49 and S50) resembled those of **12**. Detailed analysis of 2D NMR spectra (Figures S51–S53) revealed that one of the protons attached to C-1′ of **12** was replaced by a methyl formate group with resonances at *δ*_C_ 171.1 (C-1″), *δ*_H_ 3.76 (3H, s, H-2″), and *δ*_C_ 52.9 (C-2″). This moiety was confirmed by HMBC correlations from H-1′, H-2′, and H-2″ to C-1″. The absolute configuration of C-1′ was also determined by comparison of experimental and calculated ECD spectra (Figure 3B), confirming the *S* configuration of C-1′. Thus, the structure of **14** was elucidated as shown in Figure 1 and named as paraphamide B.

Five known oospolactone analogues were isolated from *Paraphoma* sp. CUGBMF180003 and identified as oospolactone (**7**) [8], 8-*O*-methyloospolactone (**8**) [9], 10-hydroxyoospolactone (**9**) [10], 9,10-dihydroxyoospolactone (**10**) [10], and oospoglycol (**11**) [11], by comparing their spectroscopic data with the respective previously reported data.

### 2.2. Biological Activity

All of the isolated compounds were subjected to tests of antibacterial activities against *Candida albicans* ATCC 10231 and *Staphylococcus aureus* ATCC 25923. Both **1** and **6** showed an inhibitory effect against *S. aureus* with minimum inhibitory concentration (MIC) values of 12.5 μg/mL; however, none of the isolates inhibited the growth of *C. albicans.*

## 3. Materials and Methods

### 3.1. General Experimental Procedures

Optical rotations (${\left[\alpha \right]}_{D}^{25}$) were measured on an Anton Paar MCP 200 Modular Circular Polarimeter (Anton Paar GmbH, Graz, Austria) in a 100 × 2 mm cell. CD spectra were recorded on an Applied Photophysics Chirascan spectropolarimeter (Applied Photophysics Ltd., Leatherhead, UK). NMR spectra were obtained on a Bruker Avance 500 spectrometer (Bruker BioSpin Corp., Billerica, MA, USA) with residual solvent peaks as references (DMSO-*d*_6_: *δ*_H_ 2.50, *δ*_C_ 39.52; acetone-*d*_6_: *δ*_H_ 2.05, *δ*_C_ 29.84; CDCl_3_: *δ*_H_ 7.26, *δ*_C_ 77.16). High-resolution ESIMS measurements were obtained on an Accurate-Mass-Q-TOF LC/MS 6520 instrument (Agilent Technologies, Santa Clara, CA, USA) in positive ion mode. HPLC was performed using an Agilent 1200 Series separation module equipped with an Agilent 1200 Series diode array and Agilent 1260 Series fraction collector (Agilent Technologies, Santa Clara, CA, USA), and an Agilent ZORBAX SB-C18 column (250 × 9.4 mm, 5 µm).

### 3.2. Microbial Material, Fermentation, Extraction, and Purification

Strain CUGBMF180003 was isolated from a mud sample collected from the intertidal zones of Shenzhen, China, and grown on a potato dextrose agar plate at 28 °C. The genomic DNA of CUGBMF180003 was extracted using the GO-GPLS-100 kit (GeneOn BioTech, Changchun, China). The ITS region was amplified using a conventional primer pair of ITS4 (5′ -TCCTCCGCTTATTGATATGC -3′) and ITS5 (5′-GGAAGTAAAAGTCGTAACAAGG -3′). PCR products were sent to Beijing Qingke Biotechnology Co., Ltd. (Beijing, China) for DNA sequencing and deposited in GenBank (accession number, MZ268156). CUGBMF180003 was identified as *Paraphoma* sp. by sequence analysis of its internal transcribed spacer (ITS) region and comparison with sequences from the GenBank database, using the BLAST program to determine an approximate phylogenetic affiliation. Alignments and calculations of sequence similarity were carried out using CLUSTAL W [12]. A neighbor-joining (NJ) tree (Figure S54) was constructed using the software package Mega version 5 [13]. Bootstrap resampling method with 1000 replicates was used in evaluating the topology of the phylogenetic trees [14]. The fungus was assigned the accession number CUGBMF180003 in the culture collection at the China University of Geosciences, Beijing. The strain CUGBMF180003 was inoculated on a potato dextrose agar plate and cultured for 5 days. Subsequently, a slit of agar with fungus was cut from the plate and inoculated into 10 1 L conical flasks, each containing solid medium consisting of rice (200 g) and artificial seawater (3.5%; 200 mL), and the flasks were incubated under static conditions at 28 °C for 30 days. The cultures were extracted three times with a mixture of EtOAc:MeOH (80:20), and the combined extracts were evaporated to dryness in vacuo. The residue was suspended in distilled water and partitioned with EtOAc. The EtOAc layer was then dried in vacuo to yield a dark residue (17.25 g). The EtOAc fraction was fractionated via vacuum liquid silica gel chromatography (80 × 80 mm column, silica gel 60 H for thin-layer chromatography) using a stepwise gradient of 80–100% hexane/CH_2_Cl_2_ and then 0–90% MeOH/CH_2_Cl_2_ to afford 12 fractions. Fraction C was fractionated on a Sephadex LH-20 column using an isocratic elution of CH_2_Cl_2_:MeOH (2:1) to yield six subfractions (C1–C6), and subfraction C6 was further fractionated by HPLC (Agilent ZORBAX SB-C18, 250 × 9.4 mm, 5 μm column, 3.0 mL/min, elution with 40% to 100% acetonitrile/H_2_O) to yield **7** (46.9 mg). Fraction D was fractionated on a Sephadex LH-20 column using an isocratic elution of CH_2_Cl_2_:MeOH (2:1), to give four subfractions (D1–D4). Subfraction D3 was further fractionated by HPLC (Agilent ZORBAX SB-C18, 250 × 9.4 mm, 5 μm column, 3.0 mL/min, elution with 40% to 100% acetonitrile/H_2_O) to yield **1** (9.5 mg) and **2** (1.3 mg). Fraction F was fractionated on a Sephadex LH-20 column using an isocratic elution of CH_2_Cl_2_:MeOH (2:1) to give six subfractions (F1–F6). Subfraction F3 was further fractionated by HPLC (Agilent ZORBAX SB-C18, 250 × 9.4 mm, 5 μm column, 3.0 mL/min, with 30% to 70% acetonitrile/H_2_O) to yield **8** (3.1 mg), **12** (7.5 mg), and **14** (1.2 mg). Subfraction F5 was further fractionated by HPLC (Agilent ZORBAX SB-C18, 250 × 9.4 mm, 5 μm column, 3.0 mL/min, elution with 30% to 60% acetonitrile/H_2_O) to yield **4** (2.7 mg), **5** (2.3 mg), **6** (8.7 mg), and **9** (10.8 mg). Fraction M was fractionated on a Sephadex LH-20 column using an isocratic elution of CH_2_Cl_2_:MeOH (2:1) to give nine subfractions (M1–M9). Subfraction M7 was further fractionated by HPLC (Agilent ZORBAX SB-C18, 250 × 9.4 mm, 5 μm column, 3.0 mL/min, elution with 30% to 72% acetonitrile/H_2_O) to yield **3** (6.3 mg) and **13** (5.8 mg). Fraction N was fractionated on a Sephadex LH-20 column using an isocratic elution of CH_2_Cl_2_:MeOH (2:1) to give seven subfractions (N1–N7). Subfraction N7 was further fractionated by HPLC (Agilent ZORBAX SB-C18, 250 × 9.4 mm, 5 μm column, 3.0 mL/min, elution with 30% to 72% acetonitrile/H_2_O) to yield **10** (1.7 mg) and **11** (6.3 mg).

7-Hydroxyoospolactone (**1**): Light yellow powder; ^1^H and ^13^C NMR data, Table 1; HRESIMS *m/z* 207.0652 [M + H]^+^ (calcd. C_11_H_11_O_4_, 207.0652).

7-Methoxyoospolactone (**2**): Light yellow powder; ^1^H and ^13^C NMR data, Table 1; HRESIMS *m/z* 221.0811 [M + H]^+^ (calcd. for C_12_H_13_O_4_, 221.0808).

7-Methoxy-9-hydroxyoospolactone (**3**): Light yellow powder; ^1^H and ^13^C NMR data, Table 1; HRESIMS *m/z* 241.0705 [M + H]^+^ (calcd. for 237.0758, C_12_H_13_O_5_, 237.0757).

10-Acetoxy-9-hydroxyoospolactone (**4**): Light yellow powder; ^1^H and ^13^C NMR data, Table 2; HRESIMS *m/z* 265.0708 [M + H]^+^ (calcd. for C_13_H_13_O_6_, 265.0707).

6-Dehydroxysescandelin (**5**): Light yellow powder; ${\left[\alpha \right]}_{D}^{25}$ −16.5 (*c* 0.20, MeOH); ^1^H and ^13^C NMR data, Table 2; HRESIMS *m/z* 207.0648 [M + H]^+^ (calcd. for C_11_H_11_O_4_, 207.0652).

Parapholactone (**6**): Light yellow powder; ${\left[\alpha \right]}_{D}^{25}$ +0.91 (*c* 0.11, MeOH); ^1^H and ^13^C NMR data, Table 2; HRESIMS *m/z* 233.0443 [M + H]^+^ (calcd. for C_12_H_9_O_5_, 233.0444).

Paraphamide A (**12**): Light yellow powder; ^1^H and ^13^C NMR data, Table 3; HRESIMS *m/z* 322.1069 [M + H]^+^ (calcd. for C_19_H_16_NO_4_, 322.1074).

Paraphamide B (**13**): Light yellow powder; ^1^H and ^13^C NMR data, Table 3; HRESIMS *m/z* 338.1022 [M + H]^+^ (calcd. for C_19_H_16_NO_5_, 338.1023).

Paraphamide C (**14**): Light yellow powder; ${\left[\alpha \right]}_{D}^{25}$ +35.0 (*c* 0.06, MeOH); ^1^H and ^13^C NMR data, Table 3; HRESIMS *m/z* 380.1130 [M + H]^+^ (calcd. for C_21_H_18_NO_6_, 380.1129).

### 3.3. Biological Activity

Compounds **1**–**14** were evaluated for their antimicrobial activities in 96-well plates according to the antimicrobial susceptibility testing standards outlined by the Clinical and Laboratory Standards Institute document M07-A7 (CLSI) [15]. Briefly, *C. albicans* ATCC 10231 was inoculated on potato dextrose agar plate and cultured for 24 hours at 35 °C. Five colonies of about 1 mm in diameter were picked and suspended in 5 mL of physiological saline. The suspension was then adjusted to approximately 10^6^ CFU/mL with RPMI 1640. For the antibacterial assay, *S. aureus* ATCC 25923 was inoculated on a Mueller–Hinton broth agar plate and cultured for 24 hours at 37 °C. Five colonies of about 1 mm in diameter were then picked and suspended in 5 mL of physiological saline. The suspension was then adjusted to approximately 10^6^ CFU/mL with Mueller–Hinton broth and 2 μL of 2-fold serial dilution of each compound (in DMSO) was added to each row in the 96-well microplate, which contained 78 μL of microbe suspension in each well. Amphotericin B and vancomycin were used as positive controls for fungi and bacteria, respectively; DMSO was used as negative control. The 96-well plates were incubated at 35 °C aerobically for 24 hours. The MIC was defined as the minimum concentration of the compound that prevented visible growth of the microbes.

## 4. Conclusions

In summary, nine new secondary metabolites, including six oospolactone analogues, 7-hydroxyoospolactone (**1**), 7-methoxyoospolactone (**2**), 7-methoxy-9-hydroxyoospolactone (**3**), 10-acetoxy-9-hydroxyoospolactone (**4**), 6-dehydroxysescandelin (**5**), and parapholactone (**6**), three compounds with a rare skeleton of isocoumarin coupled with phenylethylamine, namely paraphamide A (**12**), paraphamide B (**13**), and paraphamide C (**14**), together with five known compounds, oospolactone (**7**), 8-*O*-methoxyoospolactone (**8**), 10-hydroxyoospolactone (**9**), 9,10-dihydroxyoospolactone (**10**), and oospoglycol (**11**), were isolated from the marine-derived fungus *Paraphoma* sp. CUGBMF180003. The carbon skeleton of **12**–**14** was identified as the first example of a coupled structure of isocoumarin and phenylethylamine. Natural isocoumarin analogues display a variety of bioactivities, including inhibitory activity against α-glucosidase [16], cytotoxicity [17,18], antifungal [19] and antibacterial activities [20,21,22,23,24], and anti-influenza virus [25]. The new isocoumarin analogues **1** and **6** showed inhibitory activity against *S. aureus* with MIC values of 12.5 μg/mL, but were devoid of growth-inhibitory activity against *C. albicans* activity at a concentration of 200 μg/mL.

## Figures and Tables

**Table 1:** ^1^H (500 MHz) and ^13^C NMR (125 MHz) NMR data of **1**–**3**.

Position	1 (DMSO-*d*_6_)		2 (DMSO-*d*_6_)		3 (DMSO-*d*_6_)	
	δ_C_, Type	δ_H_ (*J* in Hz)	δ_C_, Type	δ_H_ (*J* in Hz)	δ_C_, Type	δ_H_ (*J* in Hz)
1	166.2, CO		166.2, CO		166.2, CO	
3	146.7, C		147.3, C		149.5, C	
4	108.7, C		108.6, C		110.5, C	
4a	129.6, C		130.4, C		130.0, C	
5	113.3, CH	6.93, d (8.5)	113.0, CH	6.99, d (8.5)	113.9, CH	7.12, d (8.5)
6	124.2, CH	7.31, d (8.5)	120.6, CH	7.51, d (8.5)	120.4, CH	8.50, d (8.5)
7	143.8, C		145.7, C		146.4, C	
8	148.6, C		150.2, C		150.2, C	
8a	105.9, C		105.6, C		106.0, C	
9	16.5, CH_3_	2.24, s	16.5, CH_3_	2.25, s	57.6, CH_2_	4.36, s
10	12.0, CH_3_	2.06, s	11.9, CH_3_	2.07, s	11.5, CH_3_	2.17, s
7-OH/-OMe		9.69, br s	56.1, CH_3_	3.85, s	56.1, CH_3_	3.87, s
8-OH		11.12, s		11.24, s		

**Table 2:** ^1^H (500 MHz) and ^13^C NMR (125 MHz) NMR data of **4**–**6**.

Position	4 (CDCl_3_)		5 (CDCl_3_)		6 (Acetone-*d*_6_)	
	δ_C_, Type	δ_H_ (*J* in Hz)	δ_C_, Type	δ_H_ (*J* in Hz)	δ_C_, Type	δ_H_ (*J* in Hz)
1	165.6, CO		166.4, CO		163.2, CO	
3	148.1, C		141.2, CH	7.37, s	151.1, C	
4	117.7, C		122.1, C		127.3, C	
4a	136.5, C		135.7, C		133.9, C	
5	114.9, CH	7.33, d (8.0)	113.4, CH	7.13, d (8.0)	118.9, CH	7.50, dd (7.5, 1.0)
6	137.8, CH	7.70, dd (8.0, 8.0)	137.4, CH	7.66, dd (8.0, 8.0)	137.5, CH	7.69, dd (8.5, 7.5)
7	116.7, CH	7.06, d (8.0)	116.0, CH	7.02, d (8.0)	119.0, CH	7.04, dd (8.5, 1.0)
8	162.1, C		162.4, C		163.2, C	
8a	106.9, C		106.8, C		107.8, C	
9	57.1, CH_2_	4.82, s	65.0, CH	5.06, q (6.5)	75.3, CH	5.83, dd (6.0, 3.0)
10	60.1, CH_2_	5.09, s	23.3, CH_3_	1.60, d (6.5)	39.9, CH_2_	3.06, dd (18.0, 6.0) 2.76, dd (18.0, 3.0)
11	171.8, C				196.4, C	
12	21.0, CH_3_	2.12, s				
8-OH		11.06, s		11.28, s		

**Table 3:** ^1^H (500 MHz) and ^13^C NMR (125 MHz) NMR data of **12**–**14**.

Position	12 (DMSO-*d*_6_)		13 (DMSO-*d*_6_)		14 (CDCl_3_)	
	δ_C_, Type	δ_H_ (*J* in Hz)	δ_C_, Type	δ_H_ (*J* in Hz)	δ_C_, Type	δ_H_ (*J* in Hz)
1	164.4, CO		164.4, CO		165.4, CO	
3	143.5, C		143.6, C		143.7, C	
4	122.9, C		122.9, C		123.7, C	
4a	132.4, C		132.4, C		132.2, C	
5	113.7, CH	7.11, d ( 8.0)	113.8, CH	7.11, d, 8.0	113.0, CH	6.88, d (7.5)
6	138.1, CH	7.82, dd (8.0, 8.0)	138.1, CH	7.81, dd, 8.0, 8.0	138.0, CH	7.68, dd (8.0, 7.5)
7	117.5, CH	7.14, d (8.0)	117.5, CH	7.14, d, 8.0	118.6, CH	7.12, d (8.0)
8	161.4, C		161.4, C		163.0, C	
8a	106.4, C		106.5, C		106.8, C	
9	45.6, CH_2_	4.48, s	45.6, CH_2_	4.45, s	43.7, CH_2_	4.57, d (17.5) 4.24, d (17.5)
10	160.9, CO		160.8, CO		162.1, CO	
1′	43.7, CH_2_	3.73, t (7.5)	44.1, CH_2_	3.66, t, 7.5	55.0, CH	5.34, dd (10.5, 5.5)
2′	33.8, CH_2_	2.93, t (7.5)	33.0, CH_2_	2.80, t (7.5)	35.9, CH_2_	3.51, dd (14.5, 5.5) 3.19, dd (14.5, 10.5)
3′	138.6, C		128.6, C		135.8, C	
4′	128.6, CH	7.27, d (7.0)	129.5, CH	7.04, d (8.5)	128.5, CH	7.21, d (8.0)
5′	128.4, CH	7.30, dd ( 7.0, 7.0)	115.3, CH	6.67, d (8.5)	129.1, CH	7.28, dd (8.0, 8.0)
6′	126.3, CH	7.21, t (7.0)	155.8, C		127.4, CH	7.22, t (8.0)
7′	128.4, CH	7.30, dd (7.0, 7.0)	115.3, CH	6.67, d (8.5)	129.1, CH	7.28, dd (8.0, 8.0)
8′	128.6, CH	7.27, d (7.0)	129.5, CH	7.04, d (8.5)	128.5, CH	7.21, d (8.0)
1′					171.1, CO	
2″					52.9, CH_3_	3.76, s
8-OH		10.92, s				11.04, s

## Data Availability

Data is contained within the article or supplementary material.

## Supplementary Materials

The following are available online at https://www.mdpi.com/article/10.3390/md19060313/s1, Figures S1–S53: HRESIMS, 1D and 2D NMR for compounds **1**–**6** and **12**–**14**; Figure S54: Phylogenetic tree of strain CUGBMF180003.
