# Supplementary material for: New Isocoumarin Analogues from the Marine-Derived Fungus Paraphoma sp. CUGBMF180003
Source: Mar Drugs. 2021 May 28;19(6):313. doi: 10.3390/md19060313 (PMC8227774; doi:10.3390/md19060313)
Supplement: Supplementary file 1 [file marinedrugs-19-00313-s001.zip › Supplementary_Materials.pdf]

SUPPLEMENTARY MATERIAL

New Isocoumarin Analogues from the Marine-derived Fungus *Paraphoma* sp. CUGBMF180003

Xiuli Xu [1], Jiangpeng Li [1], Kai Zhang [2], Shangzhu Wei [1], Rui Lin [1], Steven W. Polyak [3], Na Yang [4,5,*] and Fuhang Song [2,*]

[1] School of Ocean Sciences, China University of Geosciences, Beijing, 100083, P. R. China; xuxl@cugb.edu.cn (X.X); lijiangpeng@cugb.edu.cn (J.L.); weishangzhu@cugb.edu.cn (S.W.); linrui@cugb.edu.cn (R.L.)

[2] School of Light Industry, Beijing Technology and Business University, Beijing, 100048, P. R. China; zhangkai2030302071@st.btbu.edu.cn (K.Z.)

[3] School of Biological Sciences, University of South Australia, Adelaide, 5005, Australia; Steven.Polyak@unisa.edu.au (S.W.P.)

[4] CAS Key Laboratory of Experimental Marine Biology, Institute of Oceanology, Chinese Academy of Sciences, Qingdao, 266071, P. R. China

[5] Laboratory for Marine Biology and Biotechnology, Qingdao National Laboratory for Marine Science and Technology, Qingdao, 266071, P. R. China

* Correspondence: songfuhang@btbu.edu.cn (F.S.); yangna@qdio.ac.cn (N.Y.)

Table of Contents

Figure S1. HRESIMS spectrum for **1** 4
Figure S2. ^{1}H NMR spectrum (500 MHz, DMSO-d_6) of **1** 4
Figure S3. ^{13}C NMR spectrum (125 MHz, DMSO-d_6) of **1** 5
Figure S4. HSQC spectrum (500 MHz, Acetone-d_6) of **1** 5
Figure S5. ^{1}H -^{1}H COSY spectrum (500 MHz, Acetone-d_6) of **1** 6
Figure S6. HMBC spectrum (500 MHz, DMSO-d_6) of **1** 6
Figure S7. HRESIMS spectrum for **2** 7
Figure S8. ^{1}H NMR spectrum (500 MHz, DMSO-d_6) of **2** 7
Figure S9. ^{13}C NMR spectrum (125 MHz, DMSO-d_6) of **2** 8
Figure S10. HSQC spectrum (500 MHz, $CDCl_3$) of **2** 8
Figure S11. ^{1}H -^{1}H COSY spectrum (500MHz, $CDCl_3$) of **2** 9
Figure S12. HMBC spectrum (500MHz, DMSO) **2** 9
Figure S13. HRESIMS spectrum for **3** 10
Figure S14. ^{1}H NMR spectrum (500 MHz, DMSO-d_6) of **3** 10
Figure S15. ^{13}C NMR spectrum (125 MHz, DMSO-d_6) of **3** 11
Figure S16. HSQC spectrum (500 MHz, DMSO-d_6) of **3** 11
Figure S17. HMBC spectrum (500 MHz, DMSO-d_6) of **3** 12
Figure S18. HRESIMS spectrum for **4** 12
Figure S19. **^{1}**H NMR spectrum (500 MHz, $CDCl_3$) of **4** 13
Figure S20. ^{13}C NMR spectrum (125 MHz, $CDCl_3$) of **4** 13
Figure S21. HSQC spectrum (500 MHz, $CDCl_3$) of **4** 14
Figure S22. ^{1}H -^{1}H COSY spectrum (500 MHz, $CDCl_3$) of **4** 14
Figure S23. HMBC spectrum (500 MHz, $CDCl_3$) of **4** 15
Figure S24. HRESIMS spectrum for **5** 15
Figure S25. **^{1}**H NMR spectrum (500 MHz, $CDCl_3$) of **5** 16
Figure S26. ^{13}C NMR spectrum (125 MHz, $CDCl_3$) of **5** 16
Figure S27. HSQC spectrum (500 MHz, $CDCl_3$) of **5** 17
Figure S28. ^{1}H -^{1}H COSY spectrum (500 MHz, $CDCl_3$) of **5** 17
Figure S29. HMBC spectrum (500 MHz, $CDCl_3$) of **5** 18

Figure S30. HRESIMS spectrum for **6** 18
Figure S31. ^{1}H NMR spectrum (500 MHz, Acetone-d_6) of **6** 19
Figure S32. ^{13}C NMR spectrum (125 MHz, Acetone-d_6) of **6** 19
Figure S33. HSQC spectrum (500 MHz, Acetone-d_6) of **6** 20
Figure S34. ^{1}H -^{1}H COSY spectrum (500 MHz, Acetone-d_6) of **6** 20
Figure S35. HMBC spectrum (500 MHz, Acetone-d_6) of **6** 21
Figure S36. HRESIMS spectrum for **12** 21
Figure S37. ^{1}H NMR spectrum (500 MHz, DMSO-d_6) of **12** 22
Figure S38. ^{13}C NMR spectrum (125 MHz, DMSO-d_6) of **12** 22
Figure S39. HSQC spectrum (500 MHz, DMSO-d_6) of **12** 23
Figure S40. ^{1}H -^{1}H COSY spectrum (500 MHz, DMSO-d_6) of **12** 23
Figure S41. HMBC spectrum (500 MHz, DMSO-d_6) of **12** 24
Figure S42. HRESIMS spectrum for **13** 24
Figure S43. ^{1}H NMR spectrum (500 MHz, DMSO-d_6) of **13** 25
Figure S44. ^{13}C NMR spectrum (125 MHz, DMSO-d_6) of **13** 25
Figure S45. HSQC spectrum (500 MHz, DMSO-d_6) of **13** 26
Figure S46. ^{1}H -^{1}H COSY spectrum (500 MHz, DMSO-d_6) of **13** 26
Figure S47. HMBC spectrum (500 MHz, DMSO-d_6) of **13** 27
Figure S48. HRESIMS spectrum for **14** 27
Figure S49. ^{1}H NMR spectrum (500 MHz, $CDCl_3$) of **14** 28
Figure S50. ^{13}C NMR spectrum (125 MHz, $CDCl_3$) of **14** 28
Figure S51. HSQC spectrum (500 MHz, $CDCl_3$) of **14** 29
Figure S52. ^{1}H -^{1}H COSY spectrum (500 MHz, $CDCl_3$) of **14** 29
Figure S53. HMBC spectrum (500 MHz, $CDCl_3$) of **14** 30
Figure S54. Neighbor-joining phylogenetic tree of strain CUGBMF180003 31

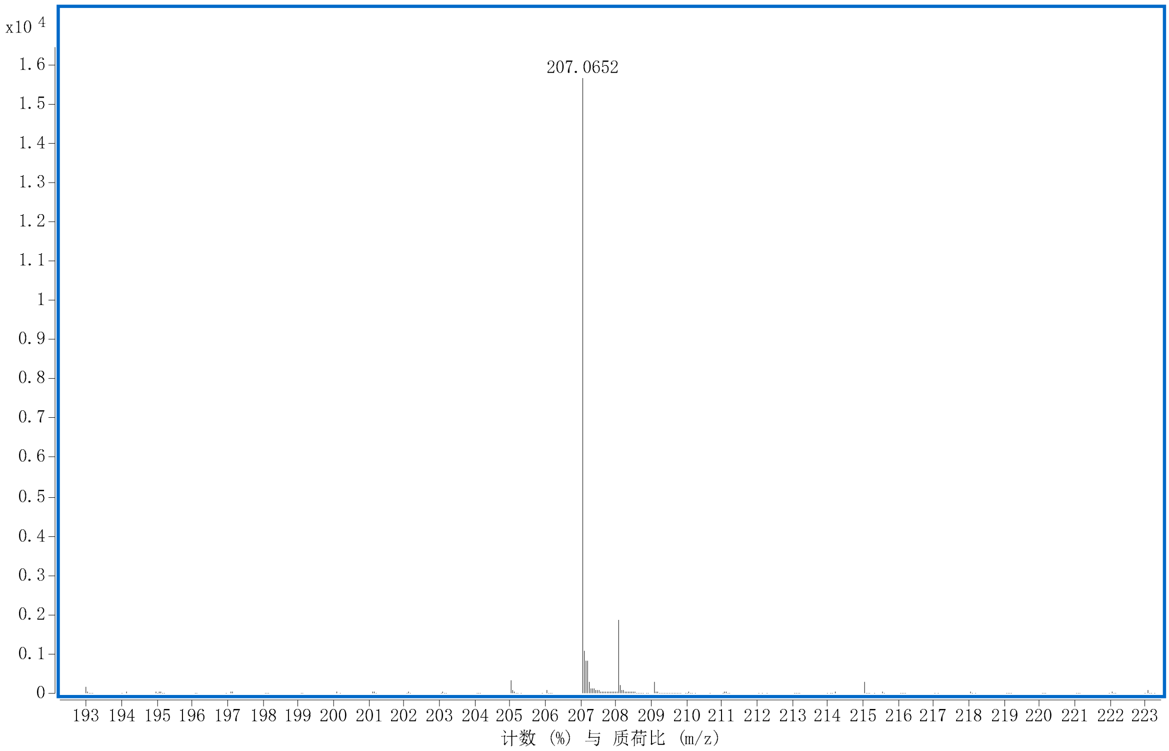

Figure S1. HRESIMS spectrum for compound **1**

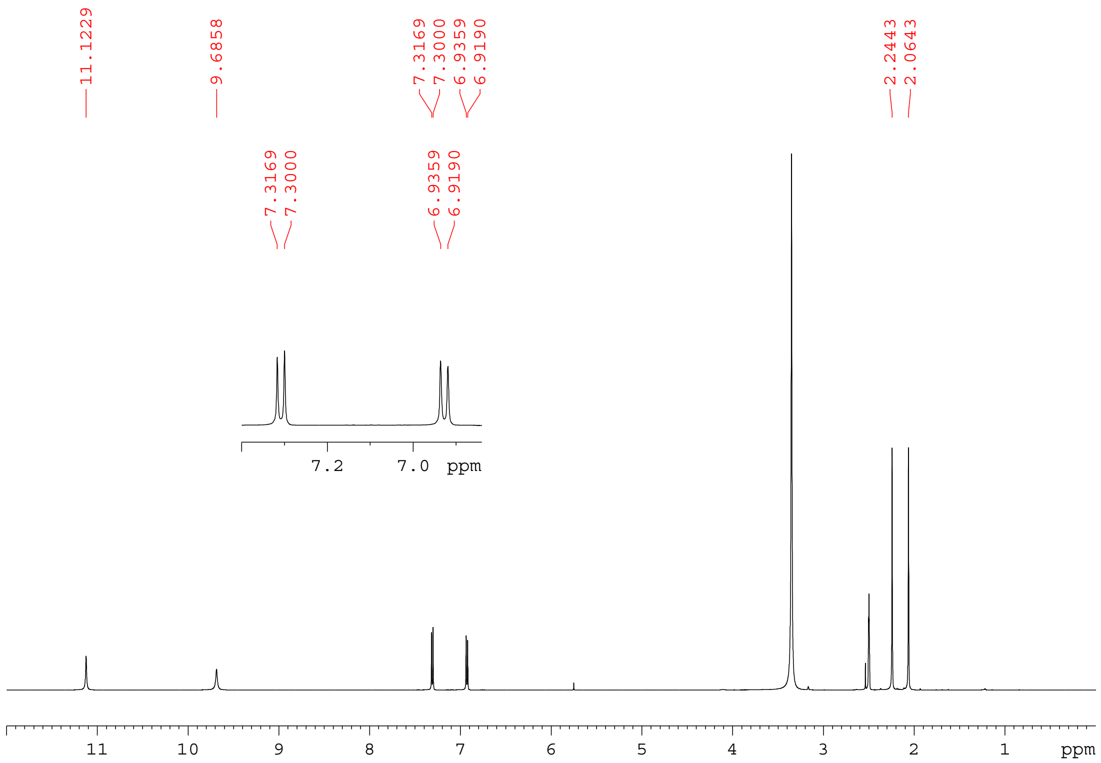

Figure S2. ^{1}H NMR spectrum (500 MHz, DMSO -d_6) of **1**

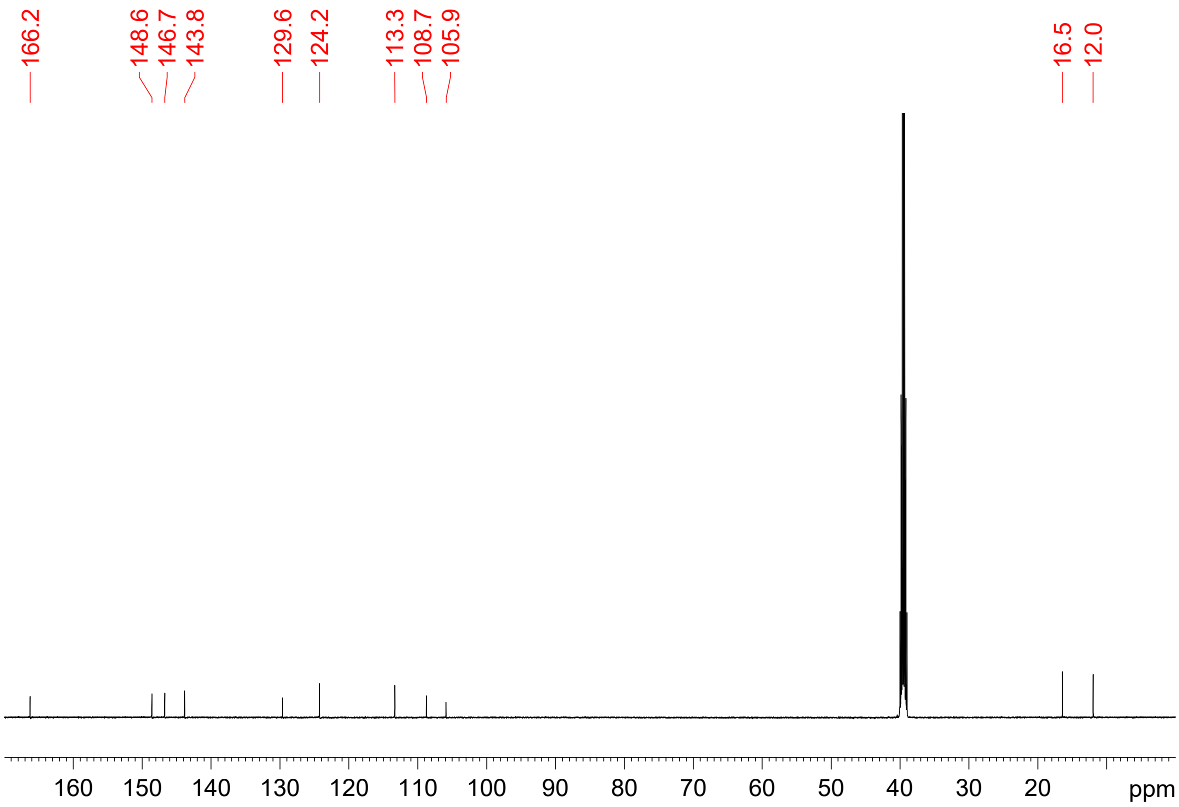

Figure S3. ^{13}C NMR spectrum (125 MHz, DMSO-d_6) of **1**

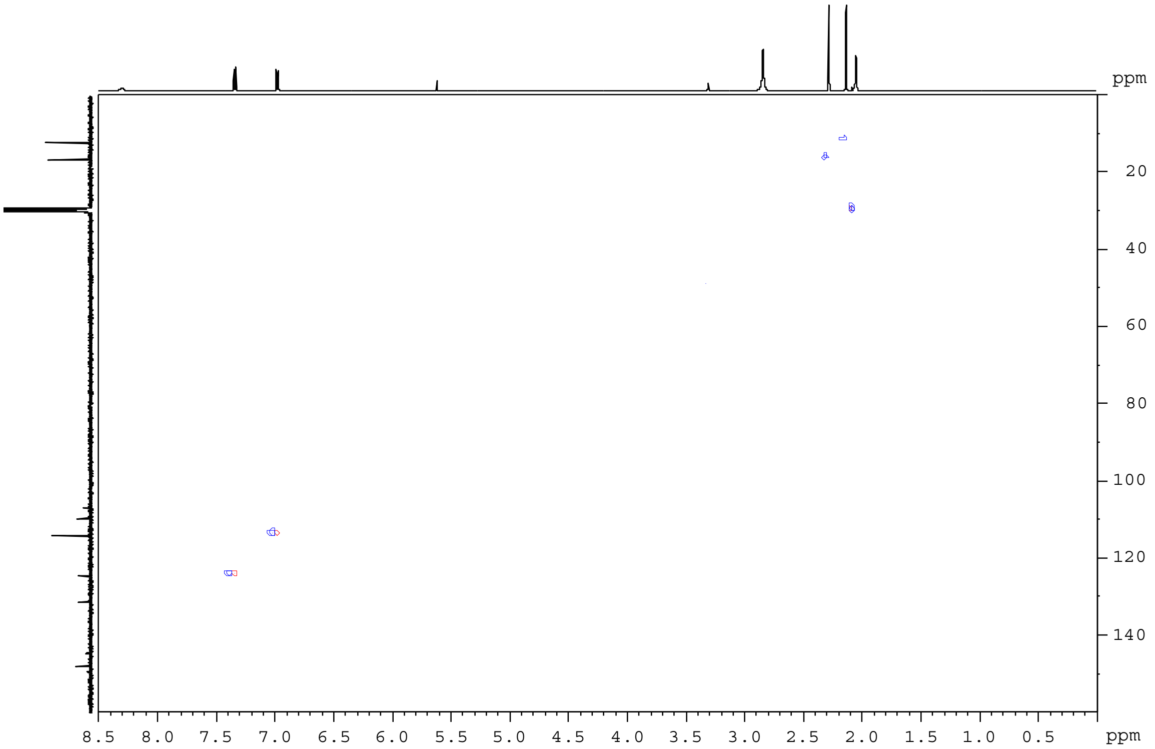

Figure S4. HSQC spectrum (500 MHz, Acetone-d_6) of **1**

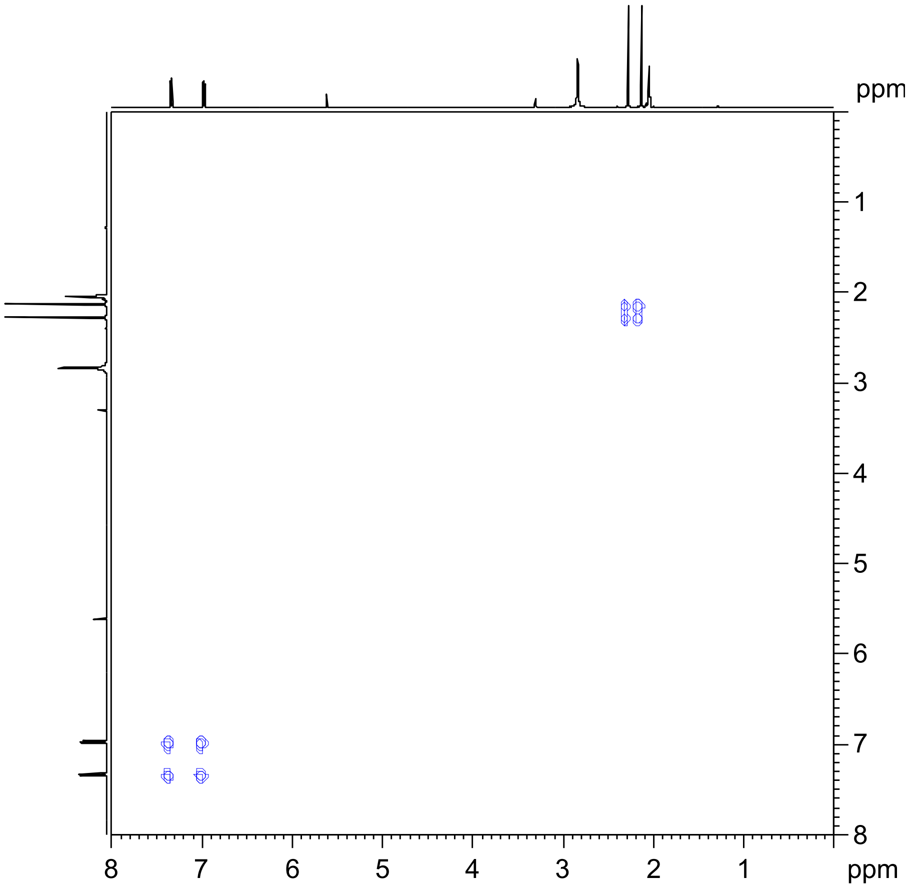

Figure S5. ^{1}H -^{1}H COSY spectrum (500 MHz, Acetone-d_6) of **1**

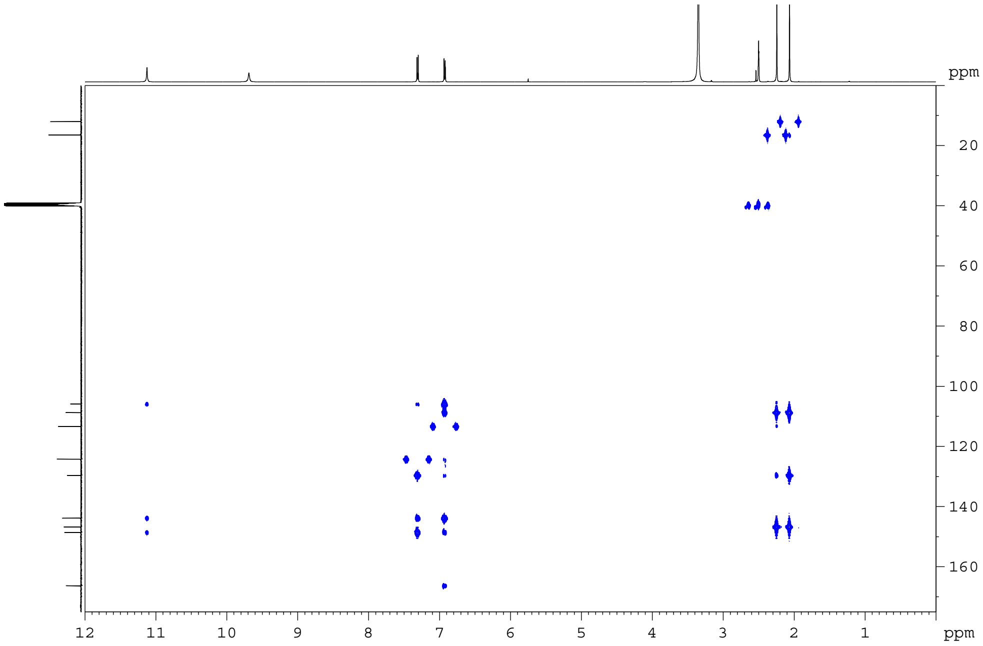

Figure S6. HMBC spectrum (500 MHz, DMSO-d_6) of **1**

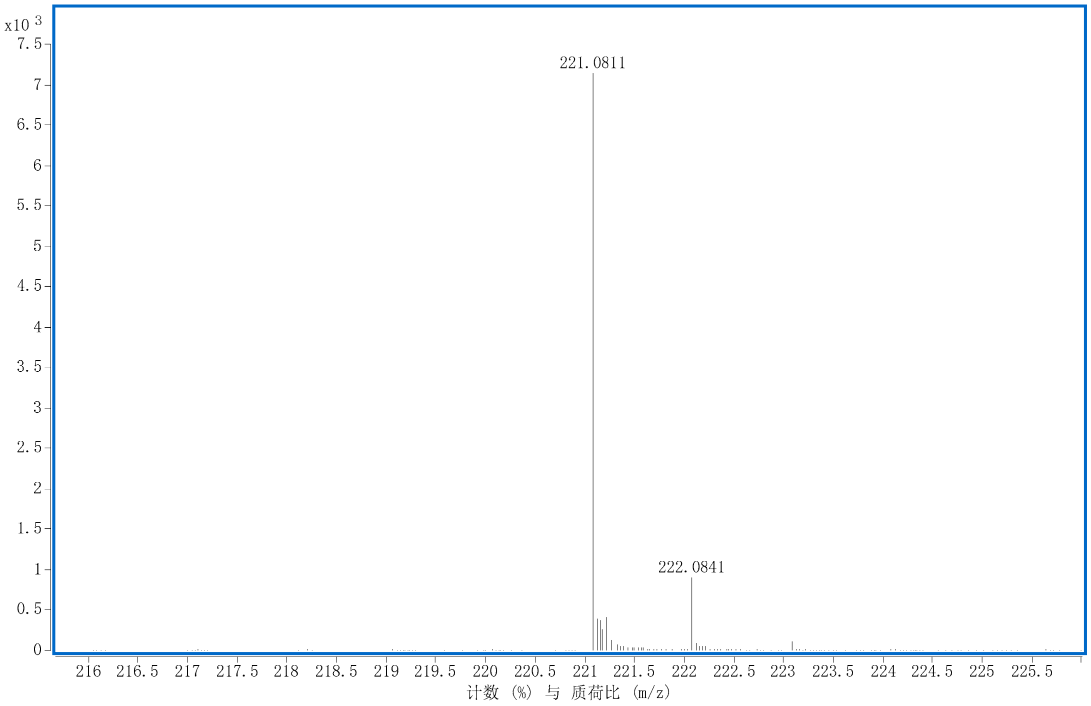

Figure S7. HRESIMS spectrum for **2**

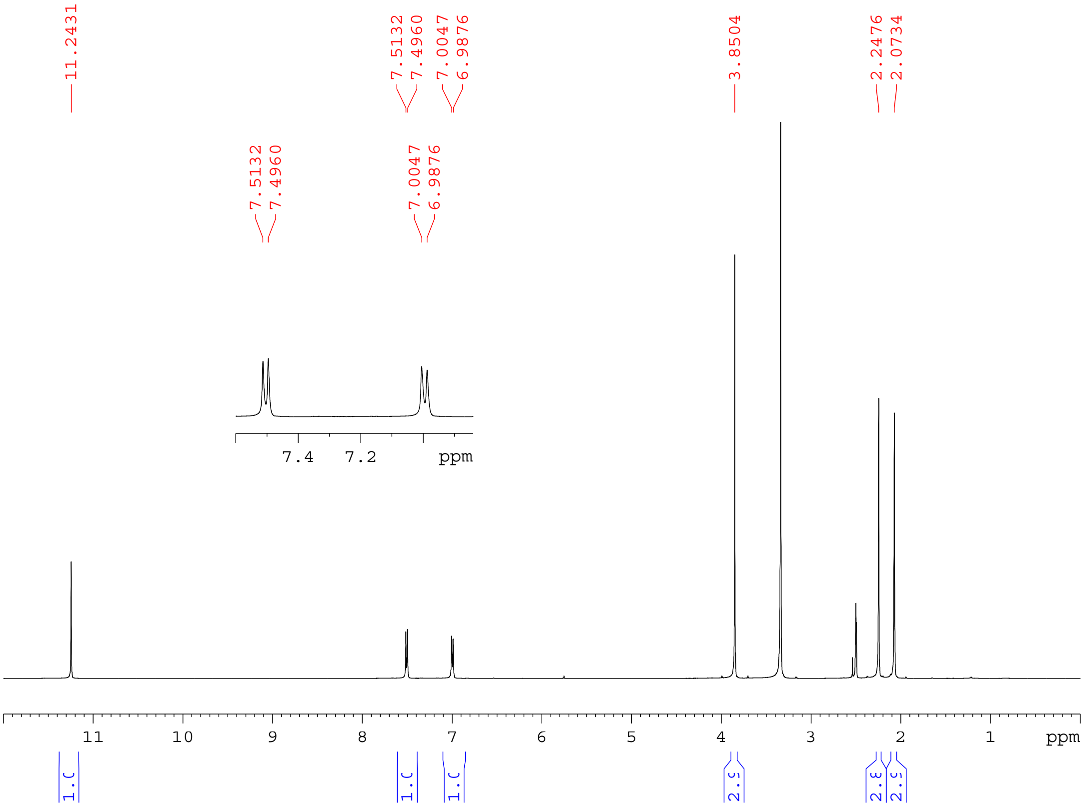

Figure S8. ^{1}H NMR spectrum (500 MHz, DMSO-d_6) of **2**

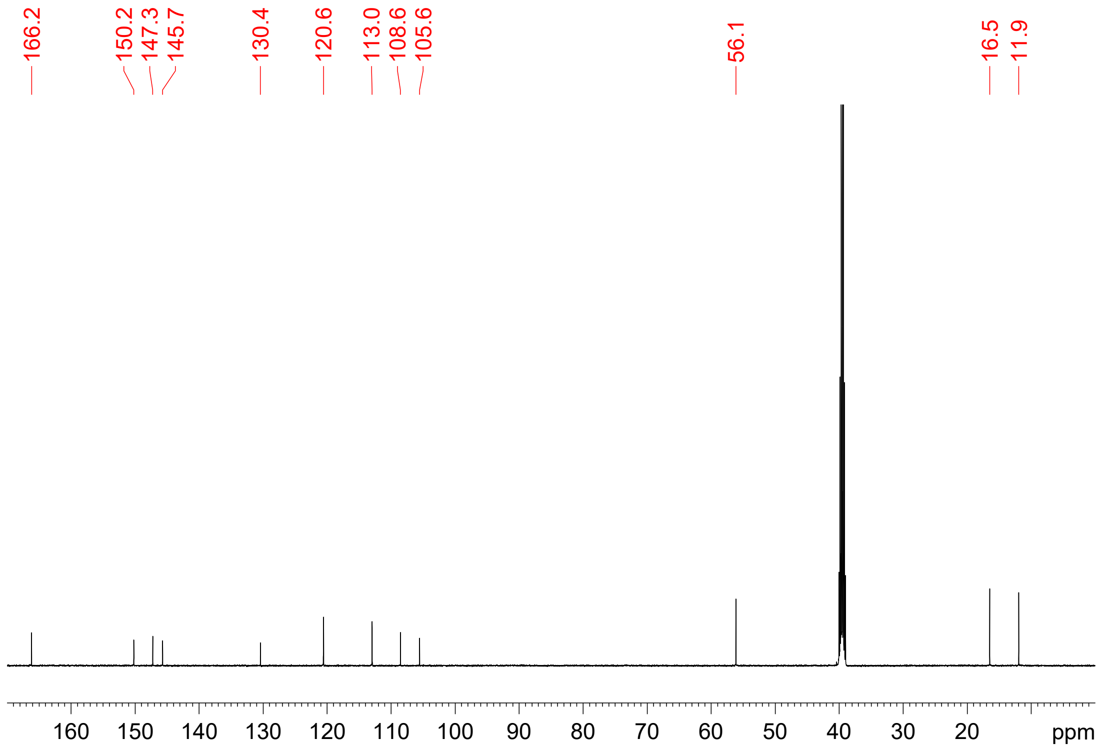

Figure S9. ^{13}C NMR spectrum (125 MHz, DMSO-d_6) of **2**

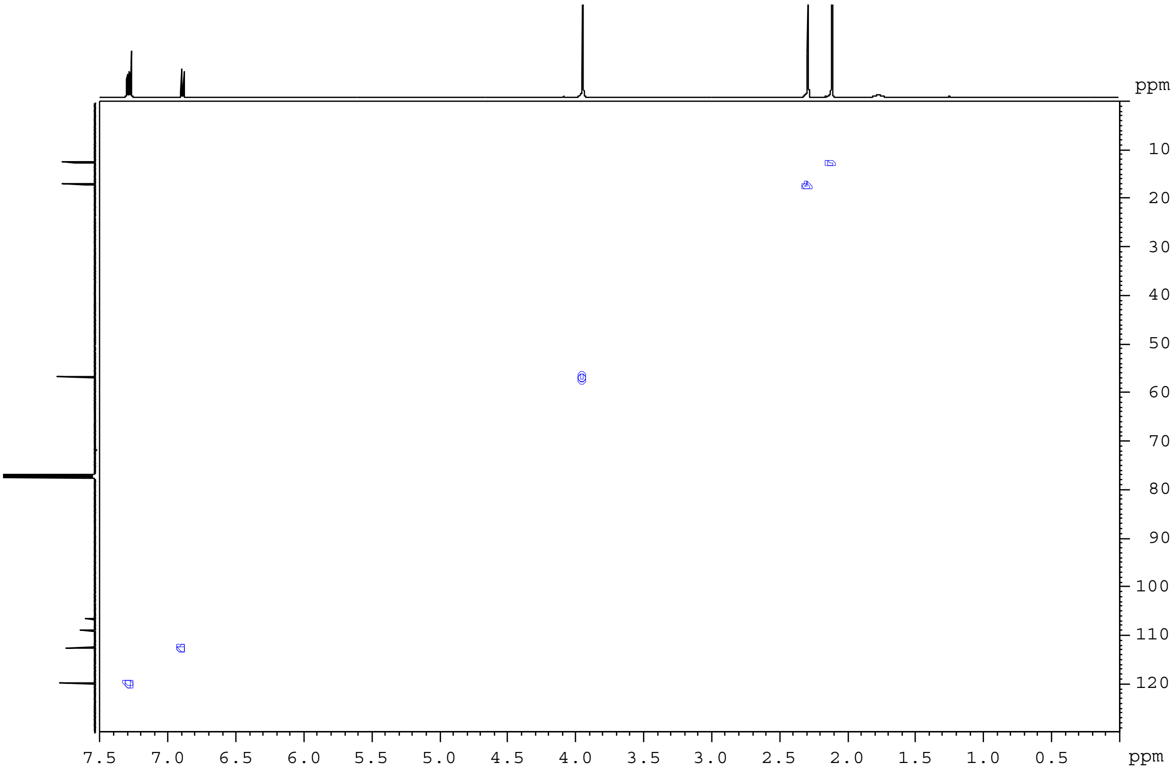

Figure S10. HSQC spectrum (500 MHz, $CDCl_3$) of **2**

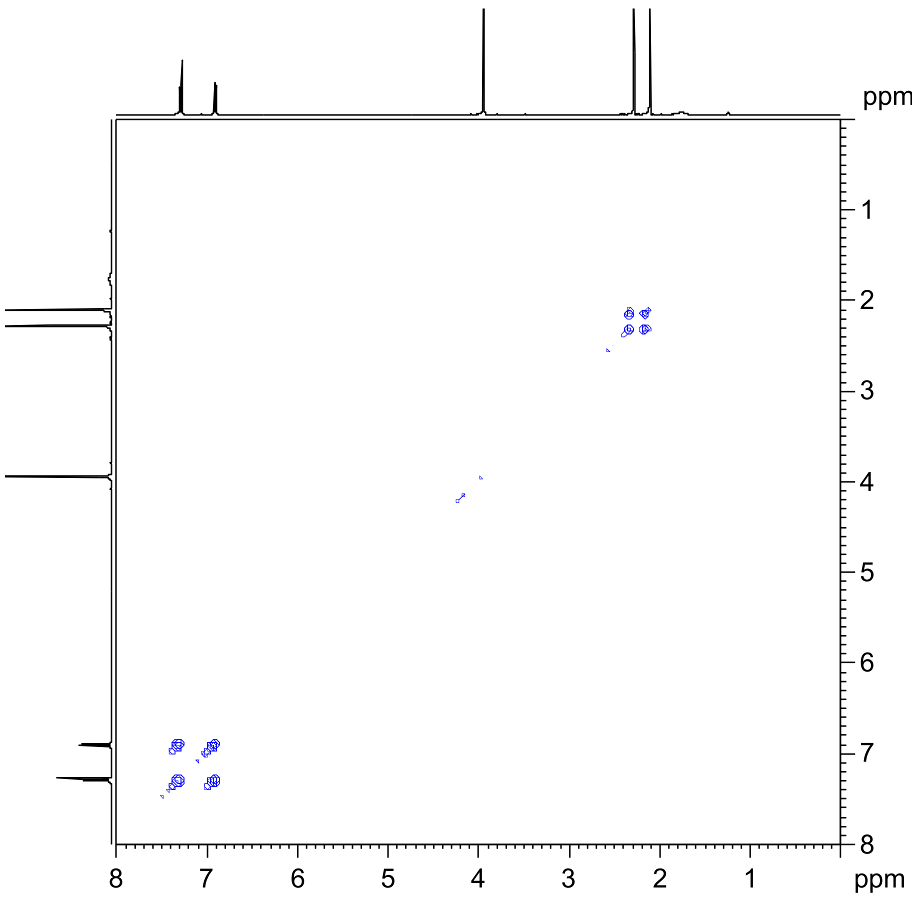

Figure S11. ^{1}H -^{1}H COSY spectrum (500MHz, $CDCl_3$) of **2**

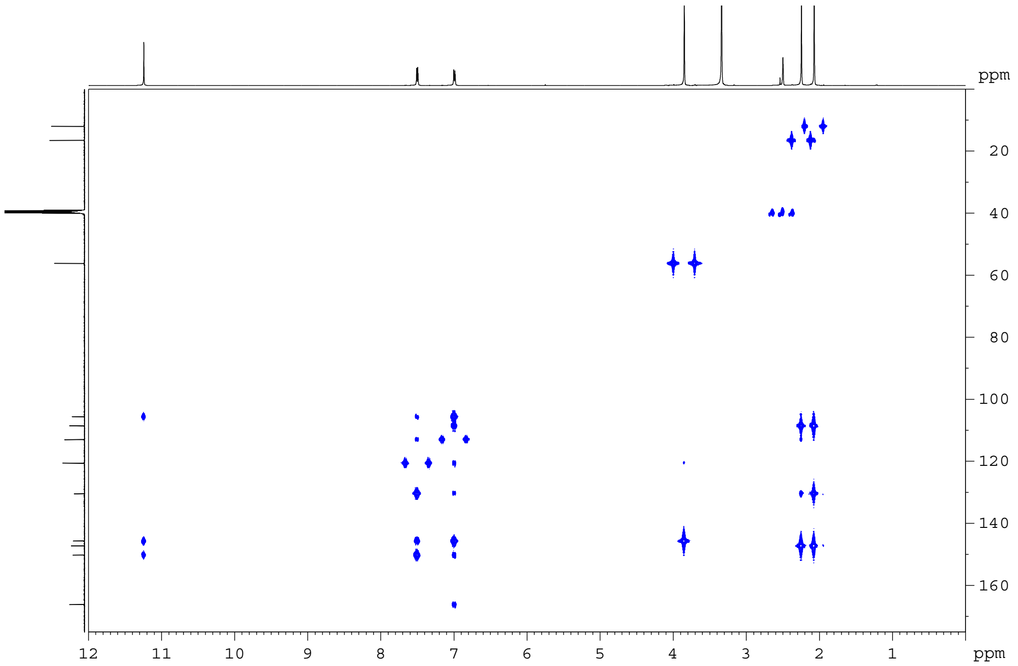

Figure S12. HMBC spectrum (500 MHz, DMSO-d_6) of **2**

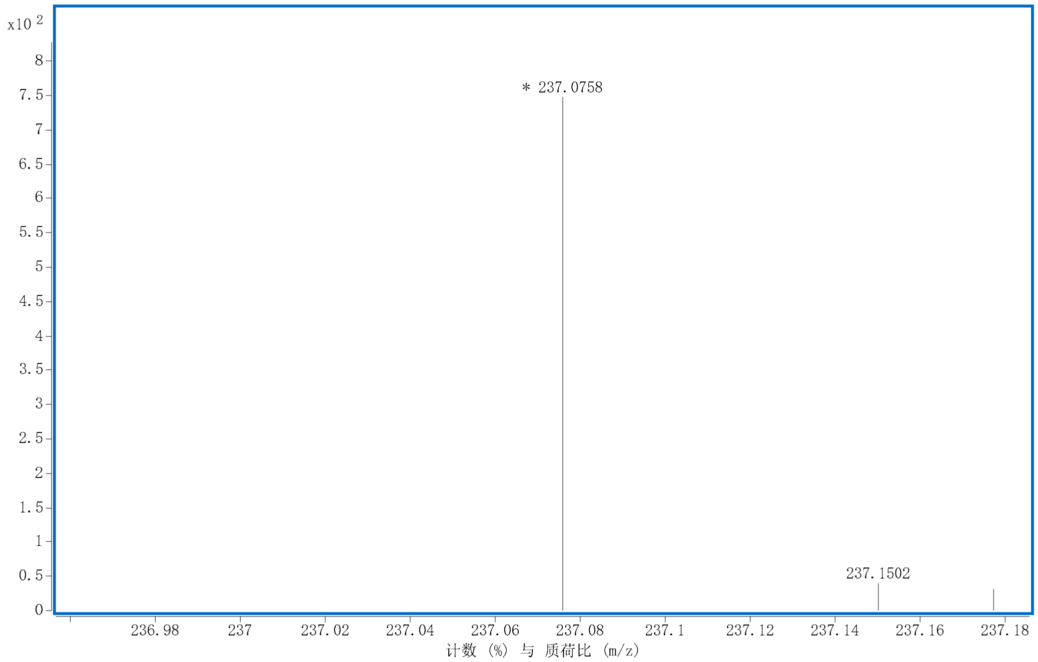

Figure S13. HRESIMS spectrum for **3**

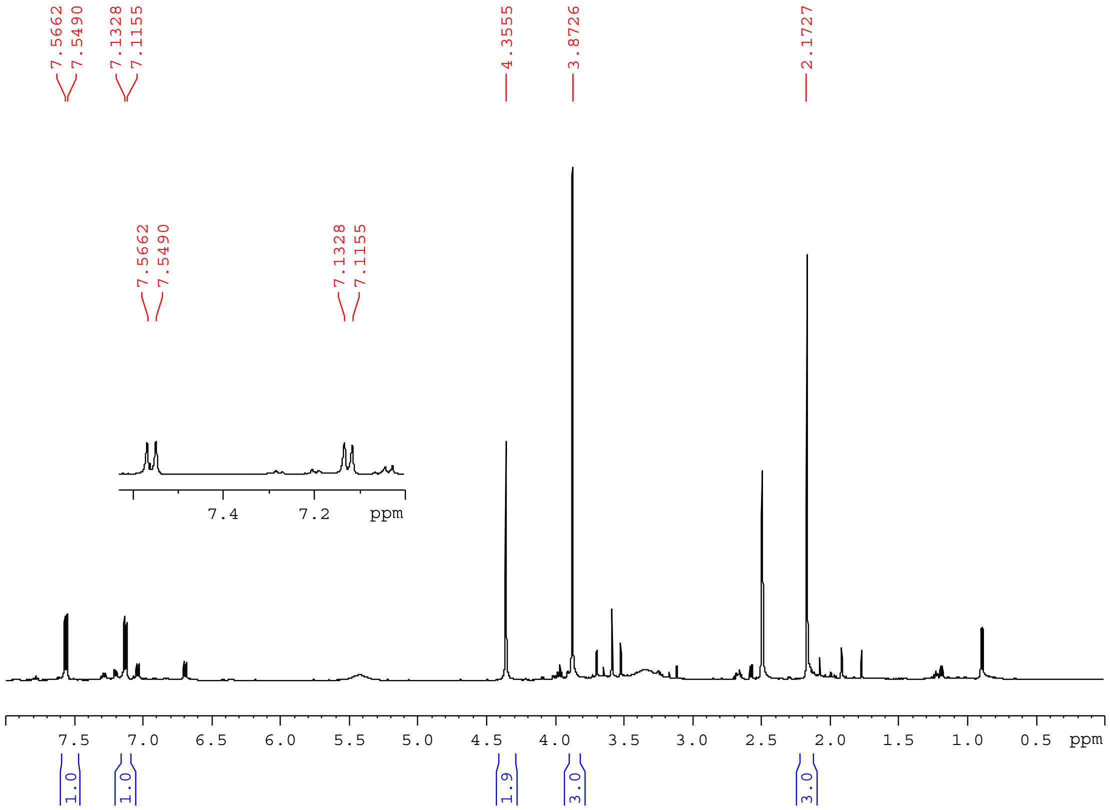

Figure S14. ^{1}H NMR spectrum (500 MHz, DMSO-d_6) of **3**

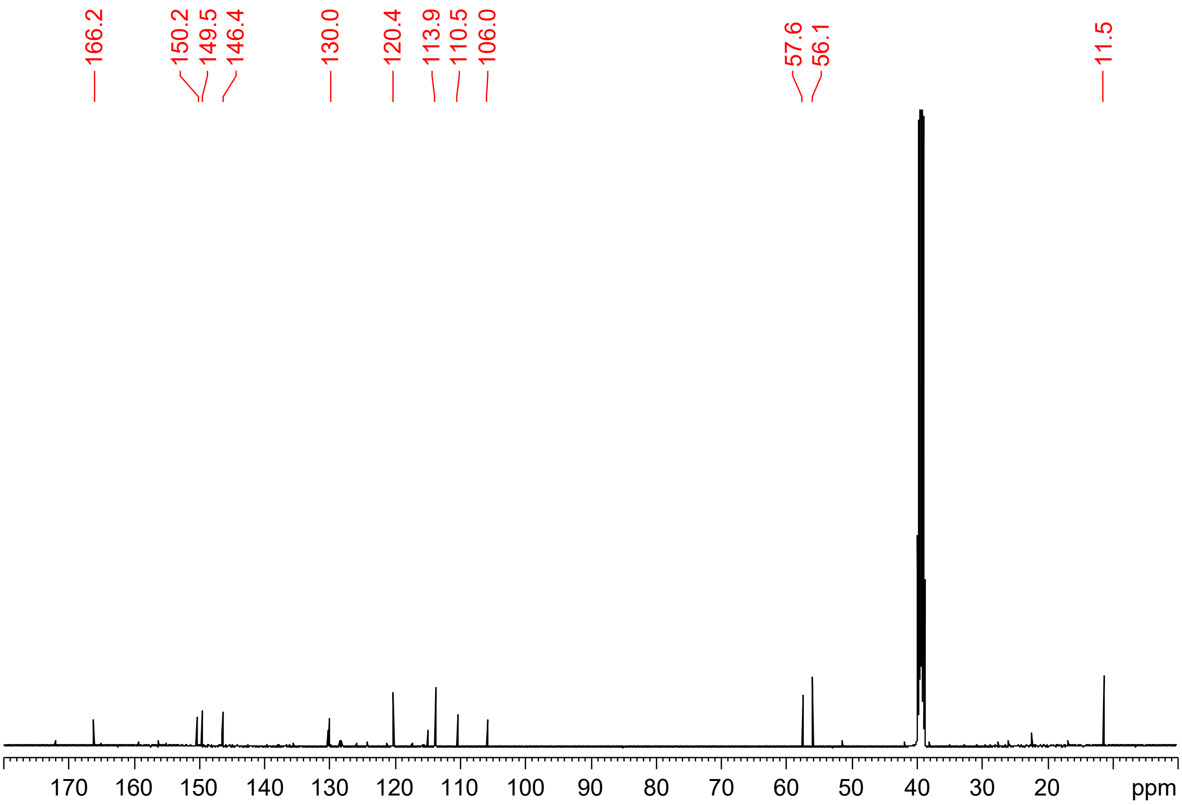

Figure S15. ^{13}C NMR spectrum (125 MHz, DMSO-d_6) of **3**

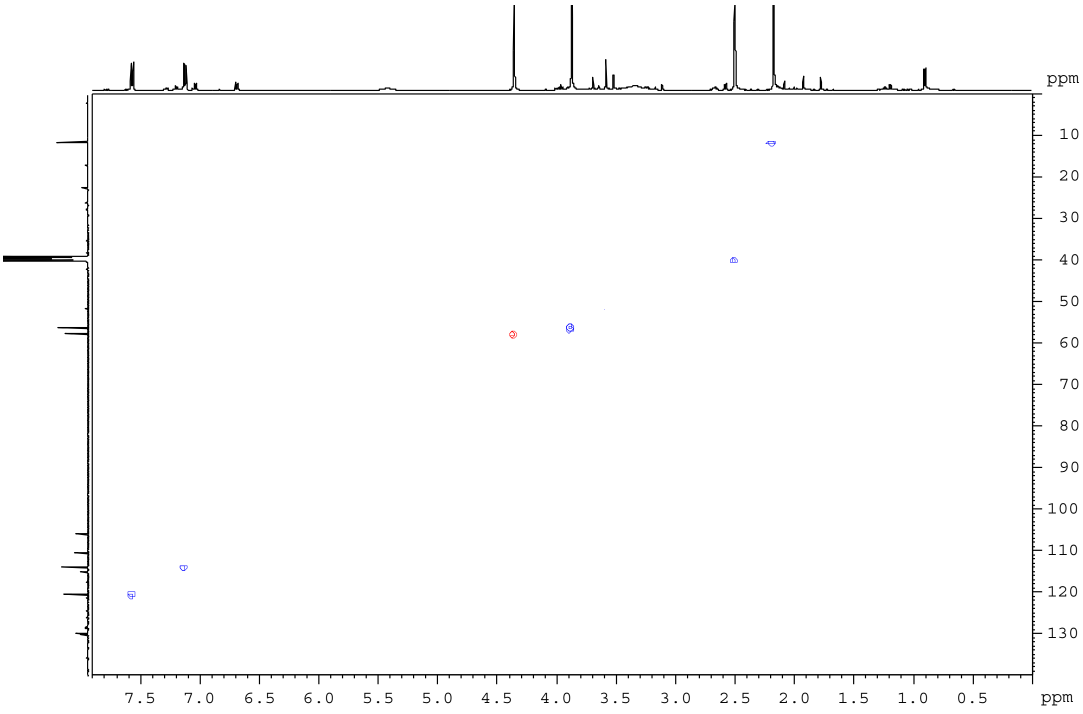

Figure S16. HSQC spectrum (500 MHz, DMSO-d_6) of **3**

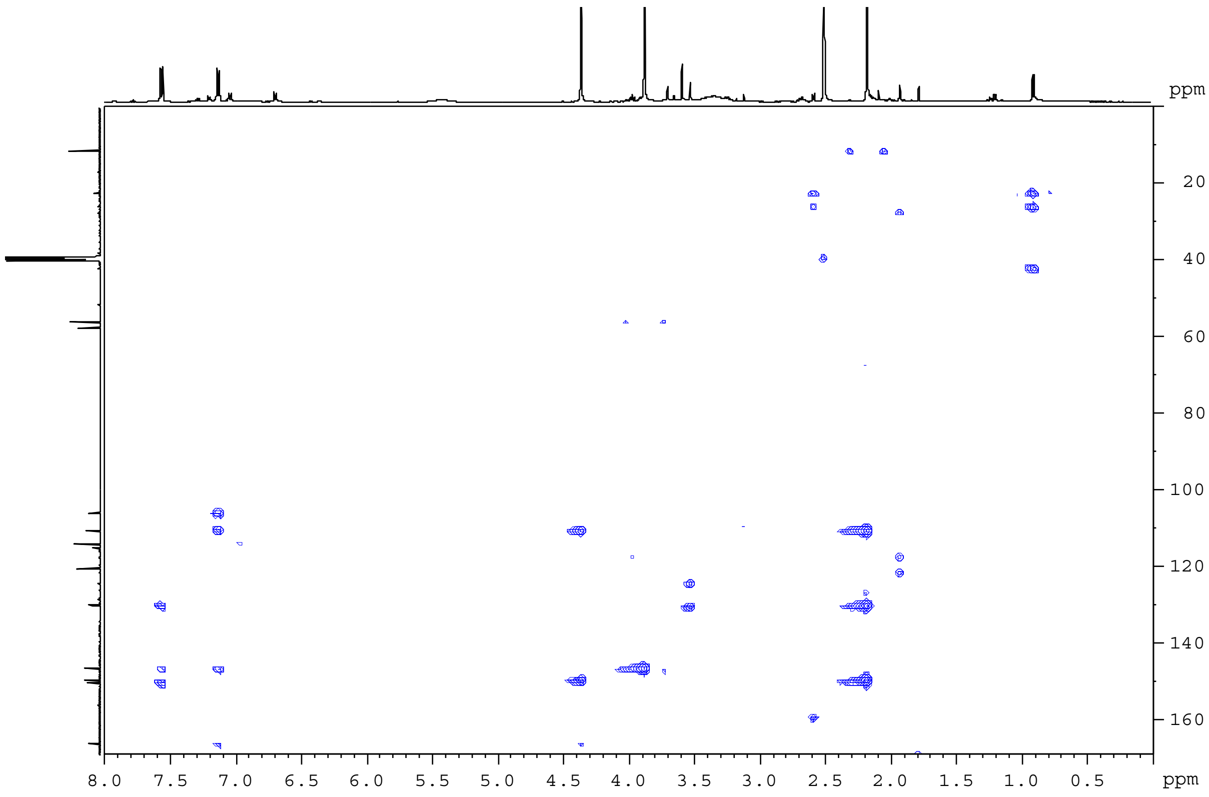

Figure S17. HMBC spectrum (500 MHz, DMSO-d_6) of **3**

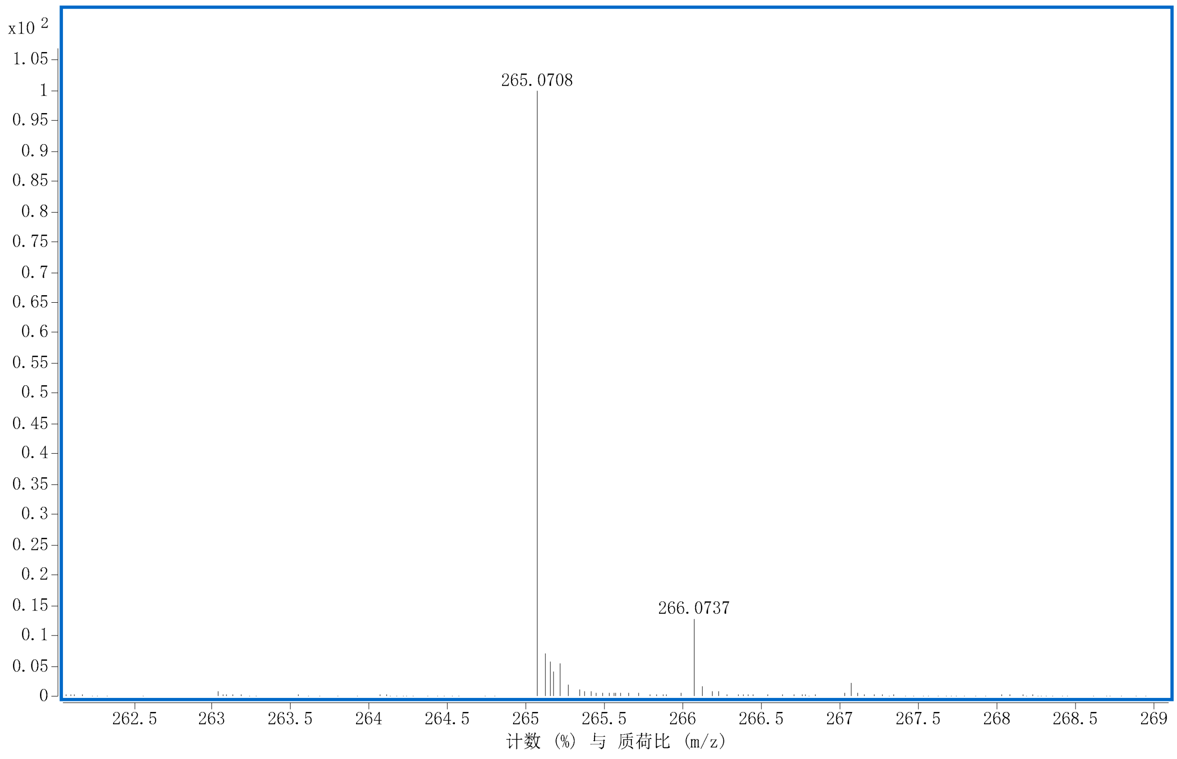

Figure S18. HRESIMS spectrum for **4**

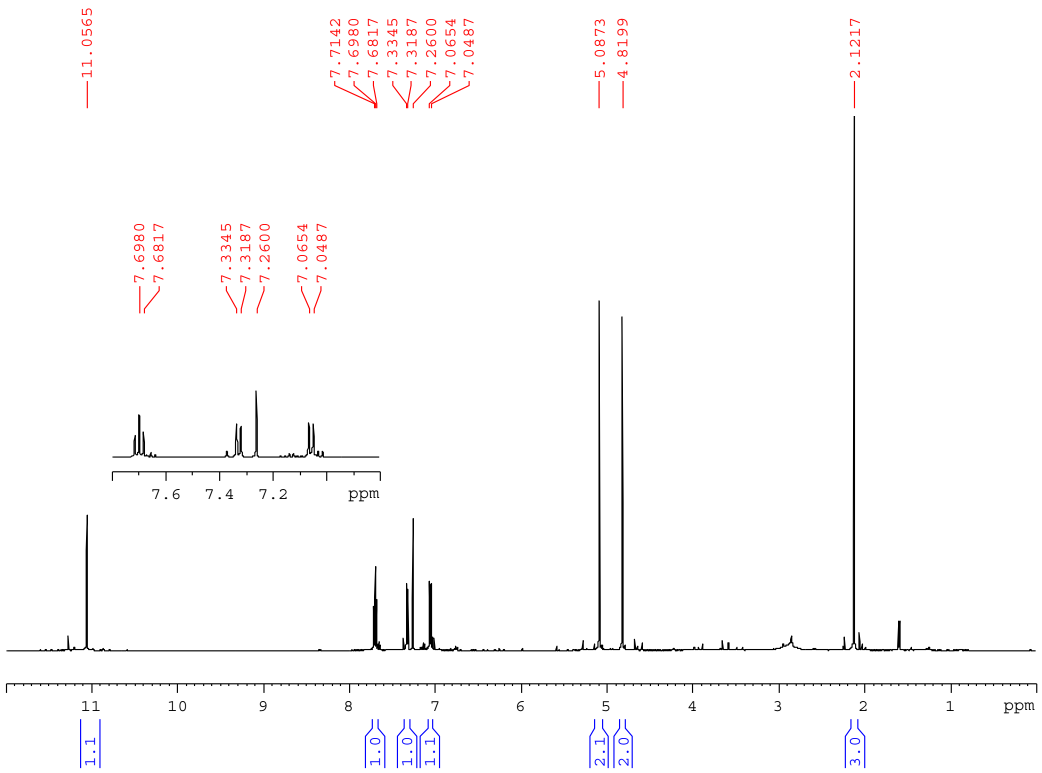

Figure S19. ^{1}H NMR spectrum (500 MHz, CDCl$_3$) of **4**

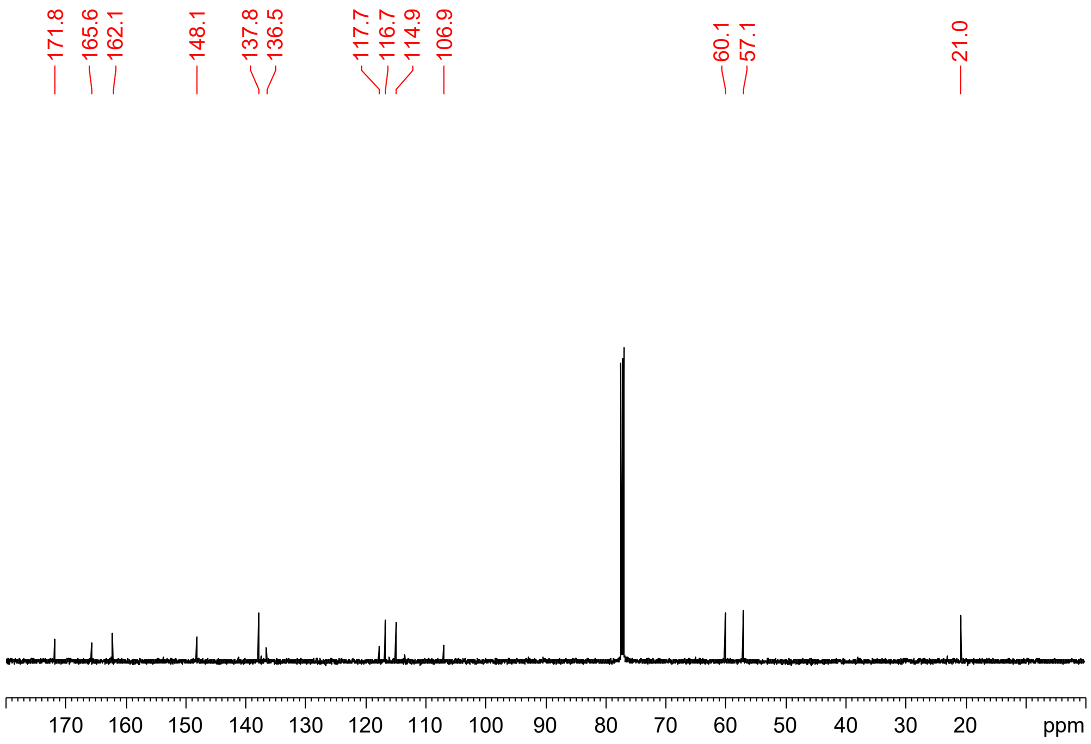

Figure S20. ^{13}C NMR spectrum (125 MHz, CDCl$_3$) of **4**

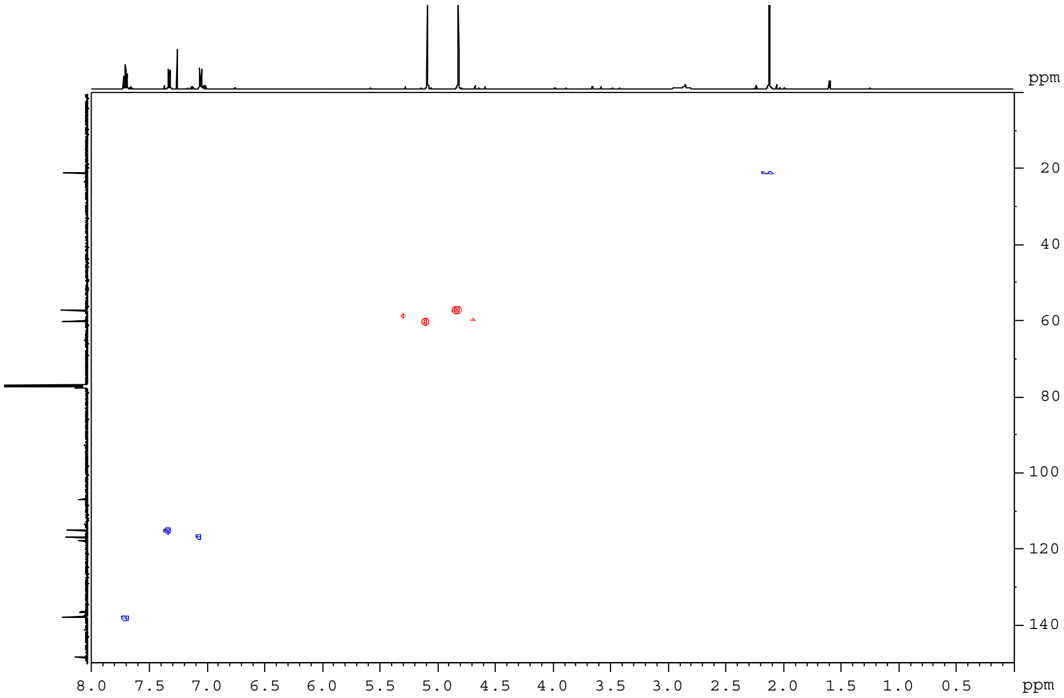

Figure S21. HSQC spectrum (500 MHz, $CDCl_3$) of **4**

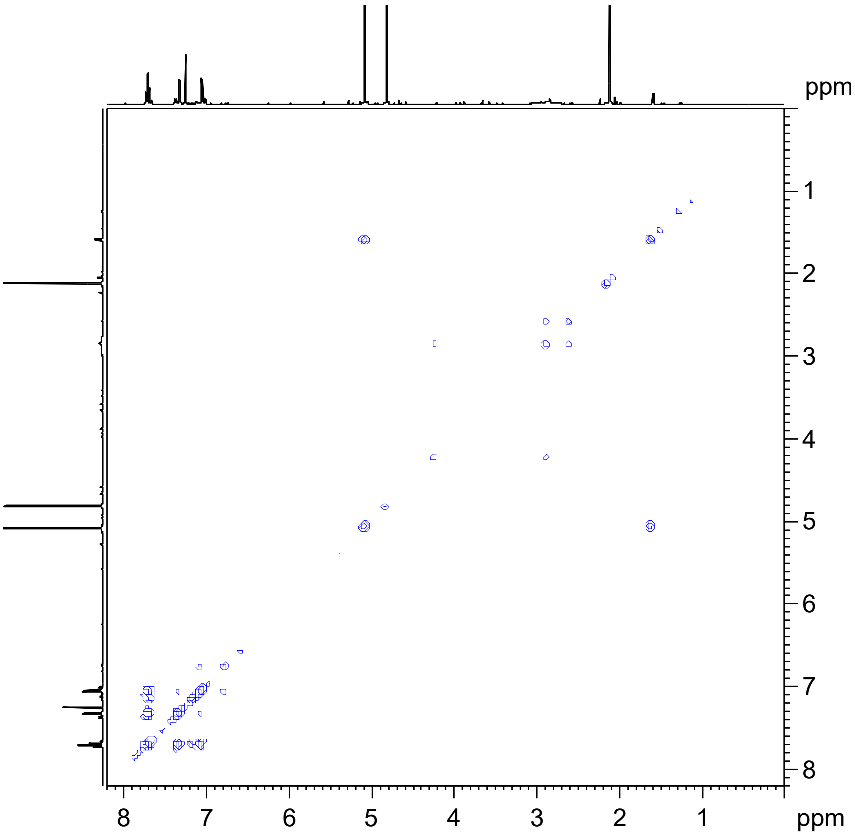

Figure S22. ^1H-^1H COSY spectrum (500 MHz, $CDCl_3$) of **4**

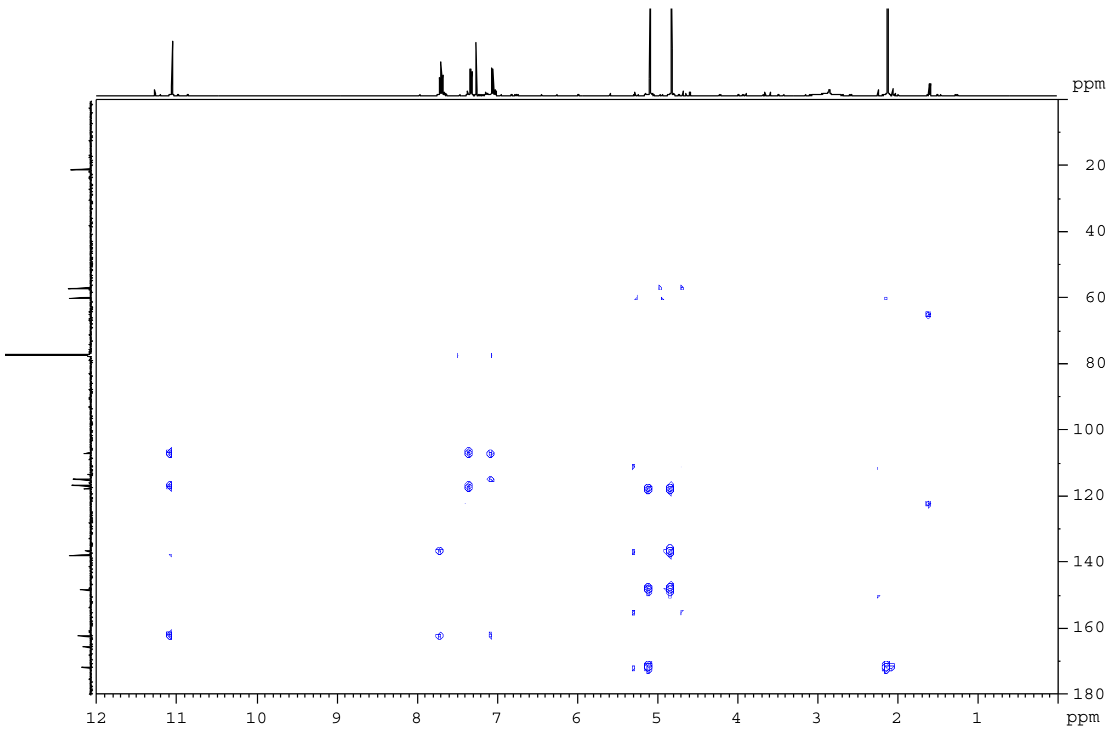

Figure S23. HMBC spectrum (500 MHz, $CDCl_3$) of **4**

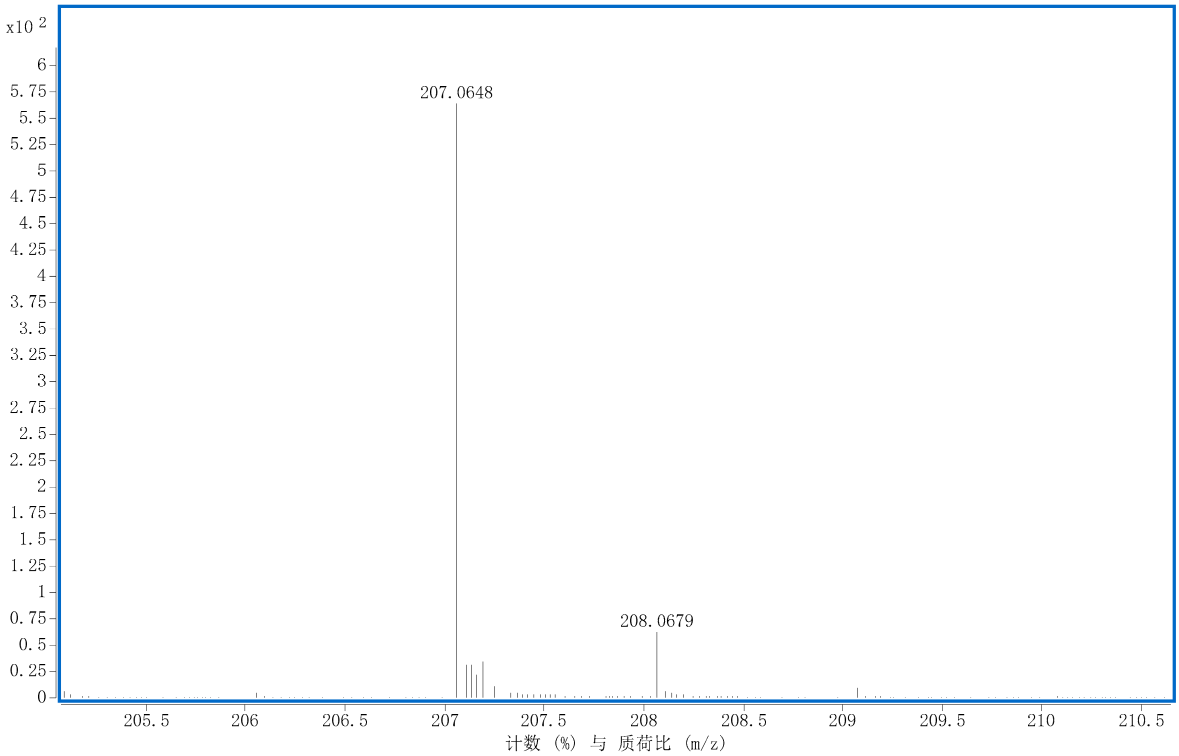

Figure S24. HRESIMS spectrum for **5**

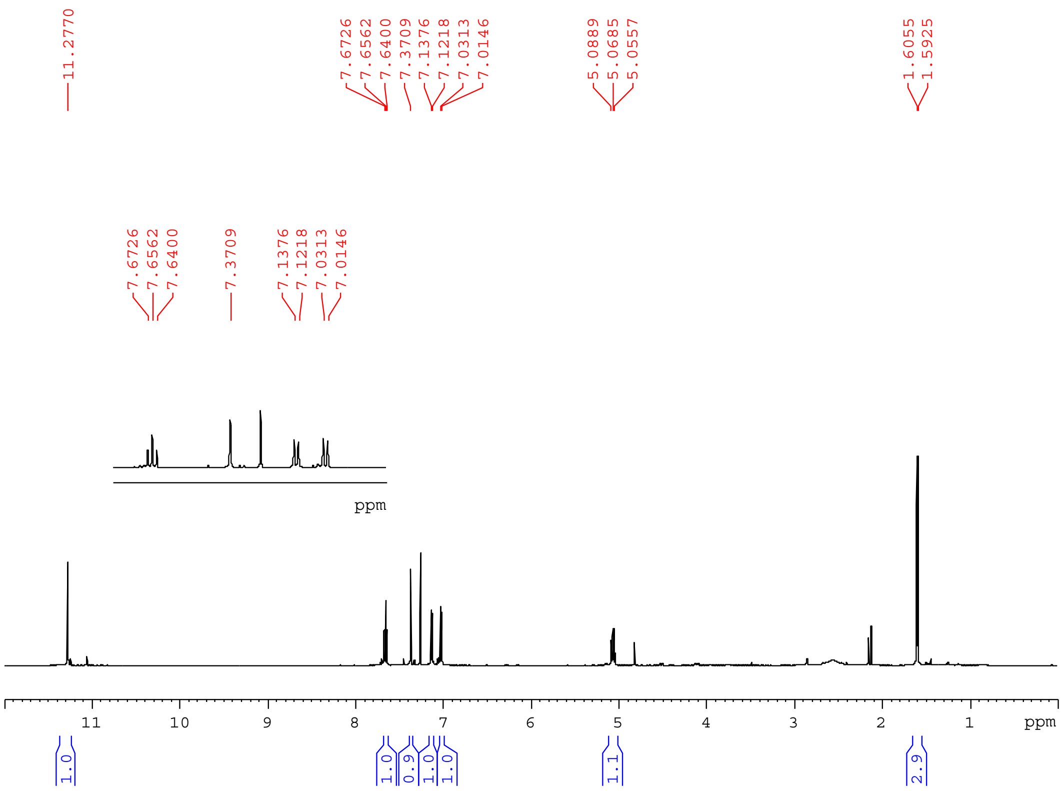

Figure S25. ^{1}H NMR spectrum (500 MHz, $CDCl_3$) of **5**

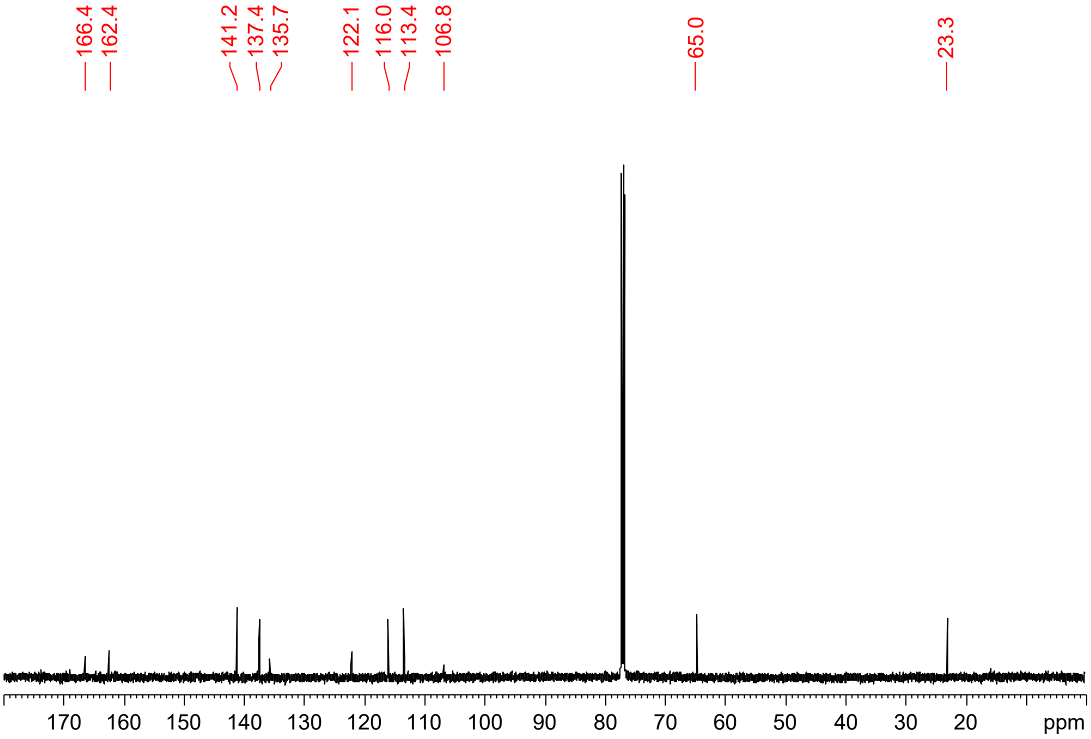

Figure S26. ^{13}C NMR spectrum (125 MHz, $CDCl_3$) of **5**

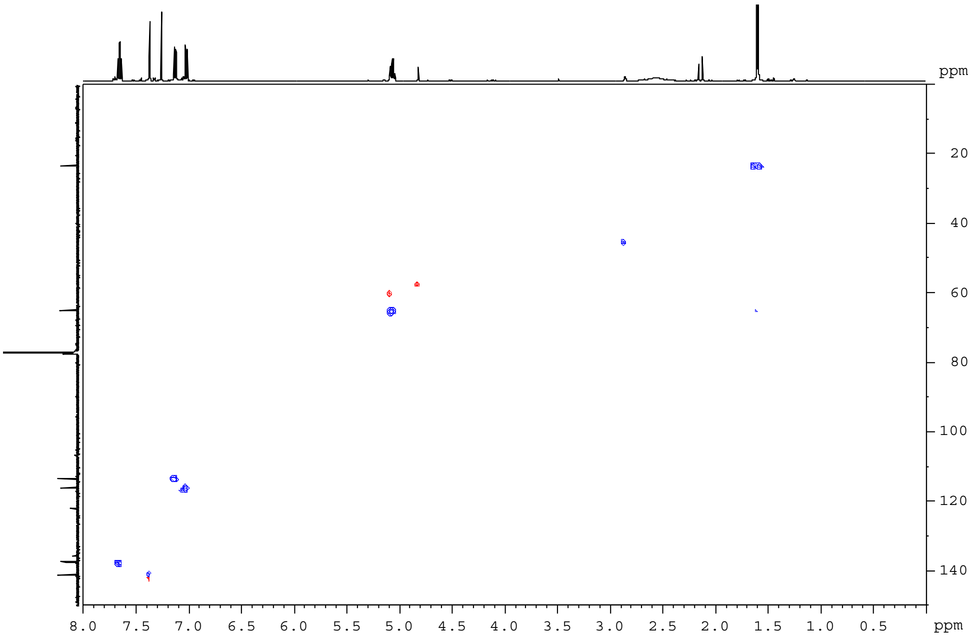

Figure S27. HSQC spectrum (500 MHz, $CDCl_3$) of **5**

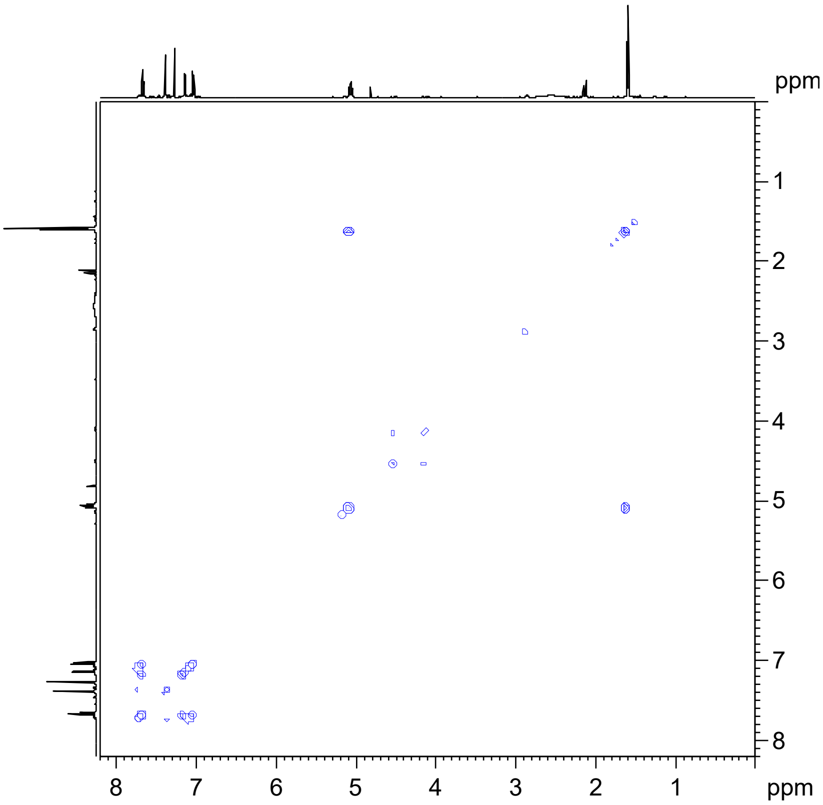

Figure S28. ^{1}H -^{1}H COSY spectrum (500 MHz, $CDCl_3$) of **5**

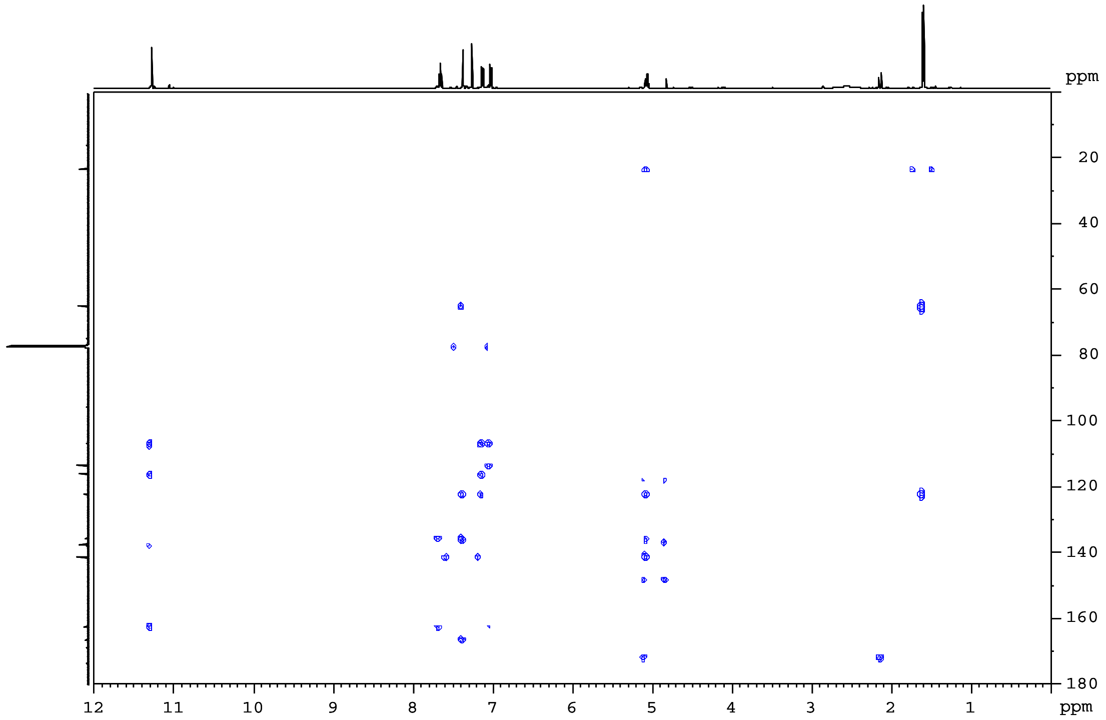

Figure S29. HMBC spectrum (500 MHz, $CDCl_3$) of **5**

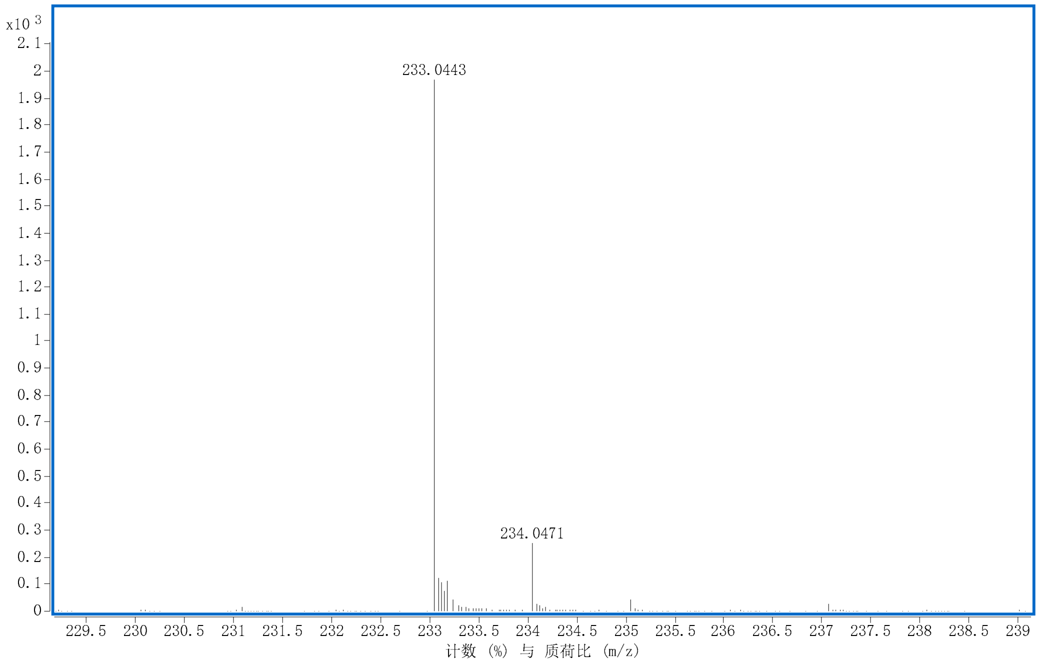

Figure S30. HRESIMS spectrum for **6**

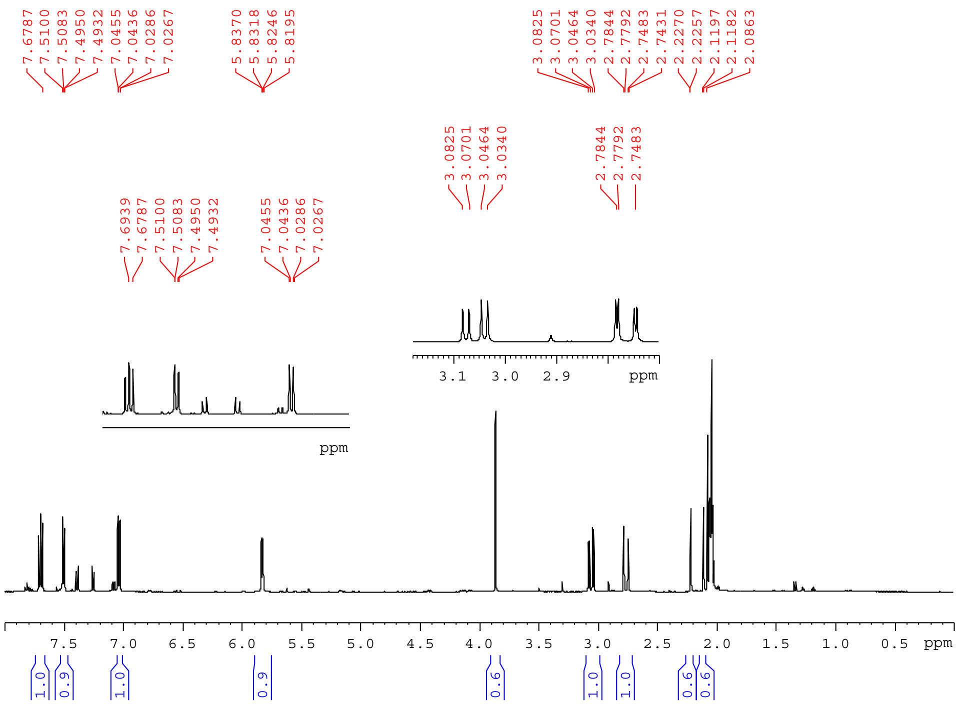

Figure S31. ^{1}H NMR spectrum (500 MHz, Acetone-d_6) of **6**

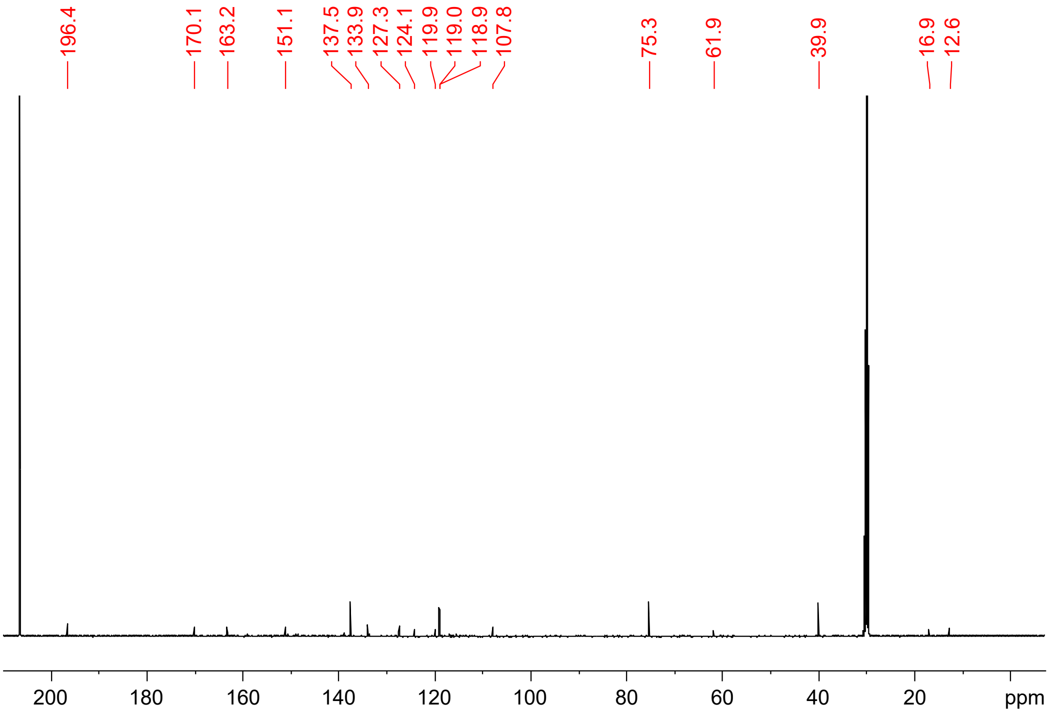

Figure S32. ^{13}C NMR spectrum (125 MHz, Acetone-d_6) of **6**

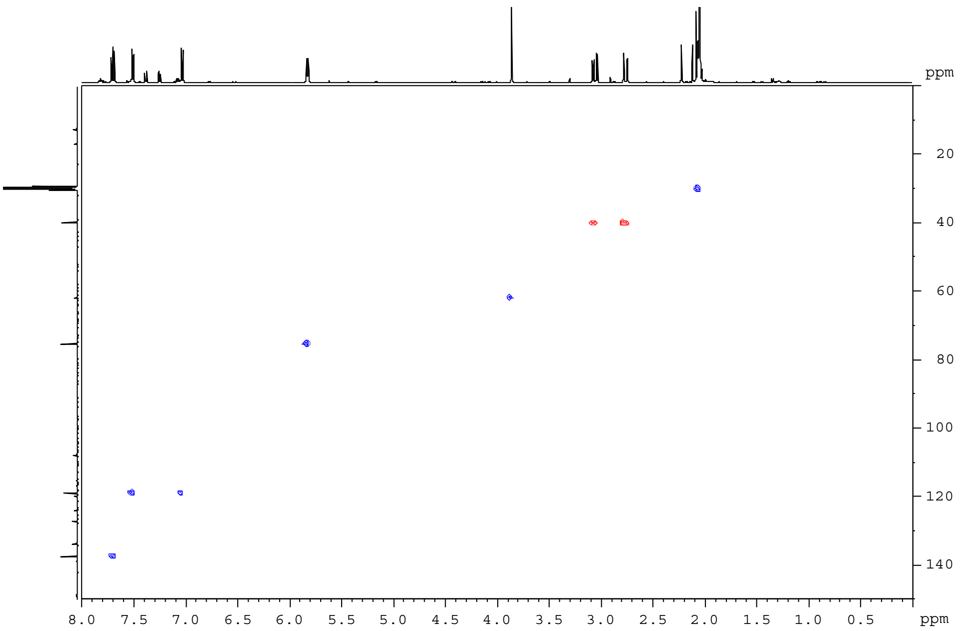

Figure S33. HSQC spectrum (500 MHz, Acetone-d_6) of **6**

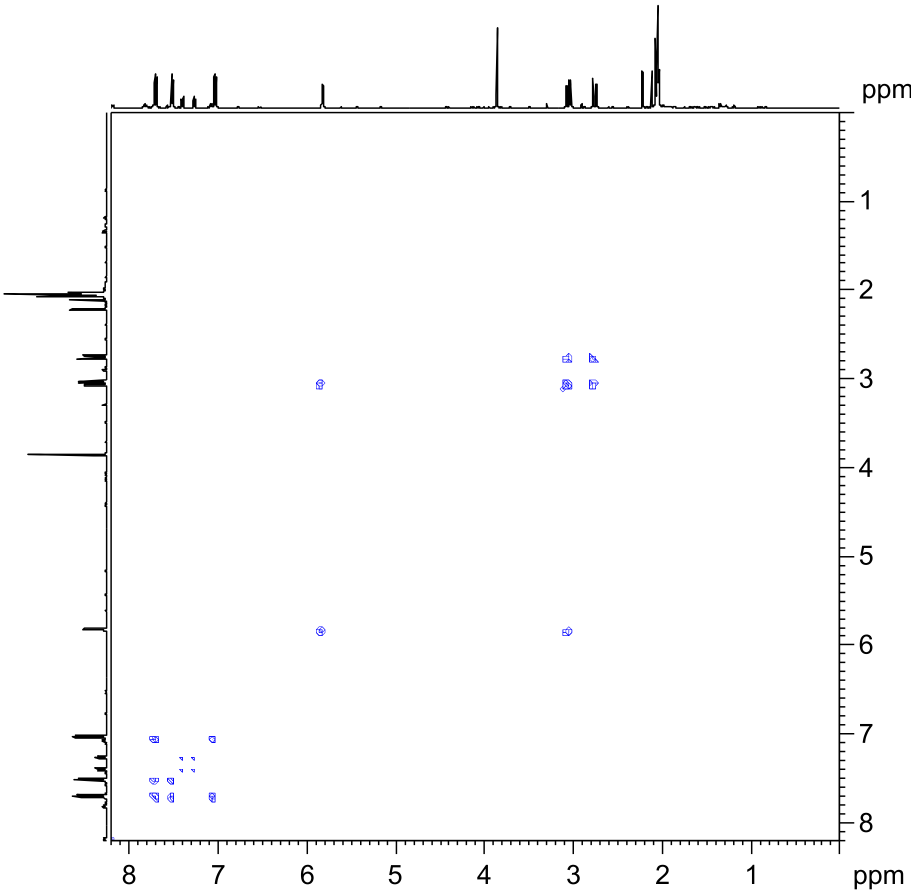

Figure S34. ^{1}H -^{1}H COSY spectrum (500 MHz, Acetone-d_6) of **6**

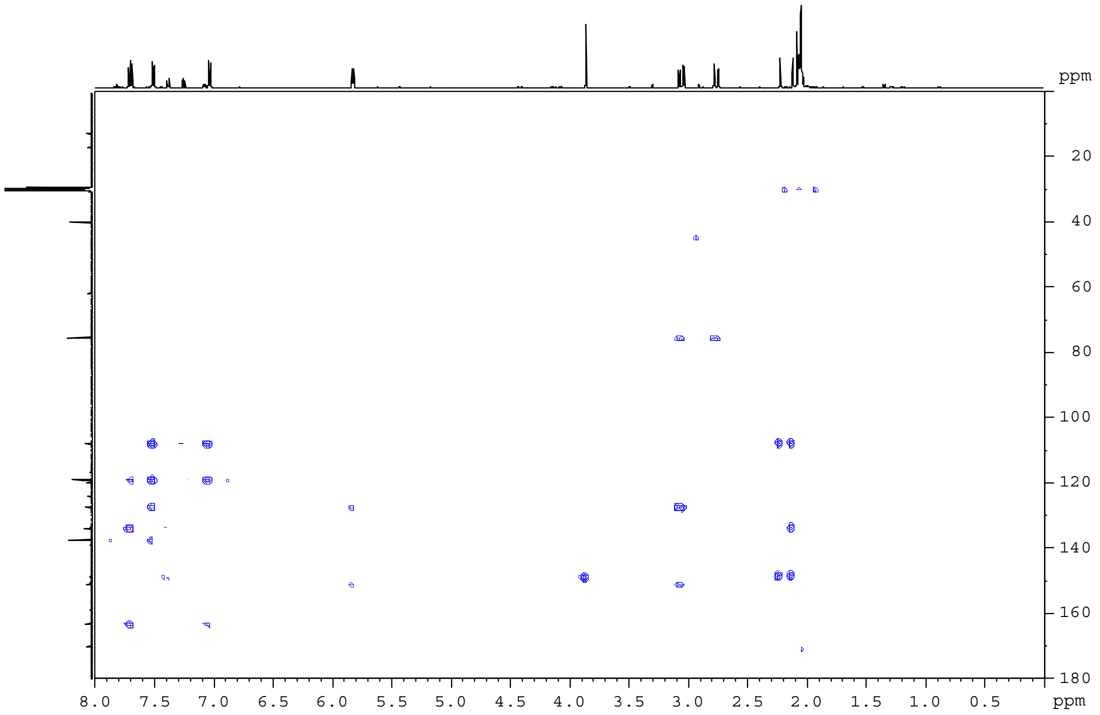

Figure S35. HMBC spectrum (500 MHz, Acetone-d_6) of **6**

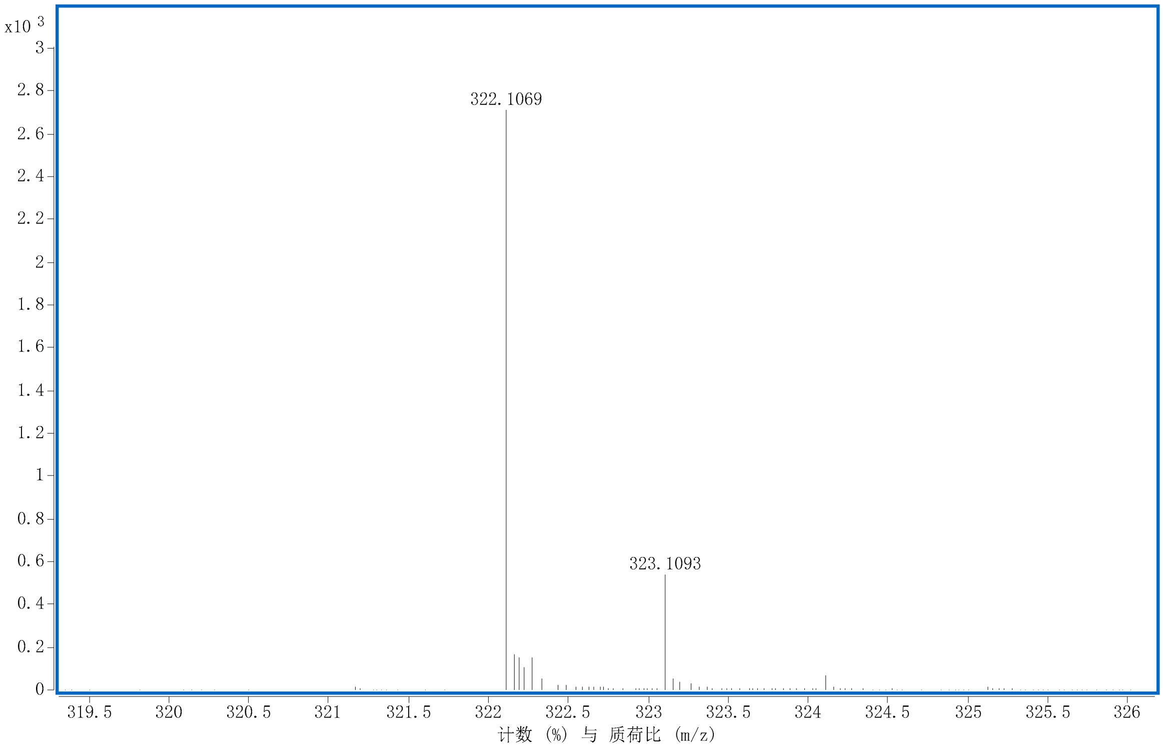

Figure S36. HRESIMS spectrum for **12**

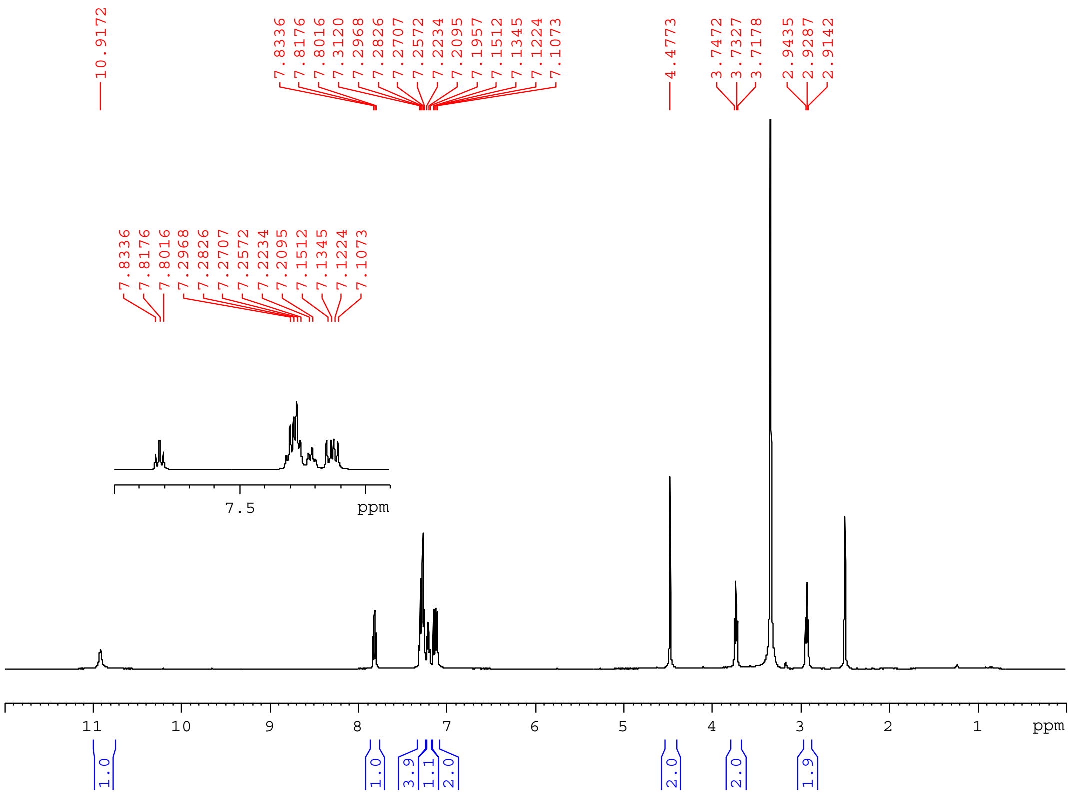

Figure S37. ^{1}H NMR spectrum (500 MHz, DMSO-d_6) of **12**

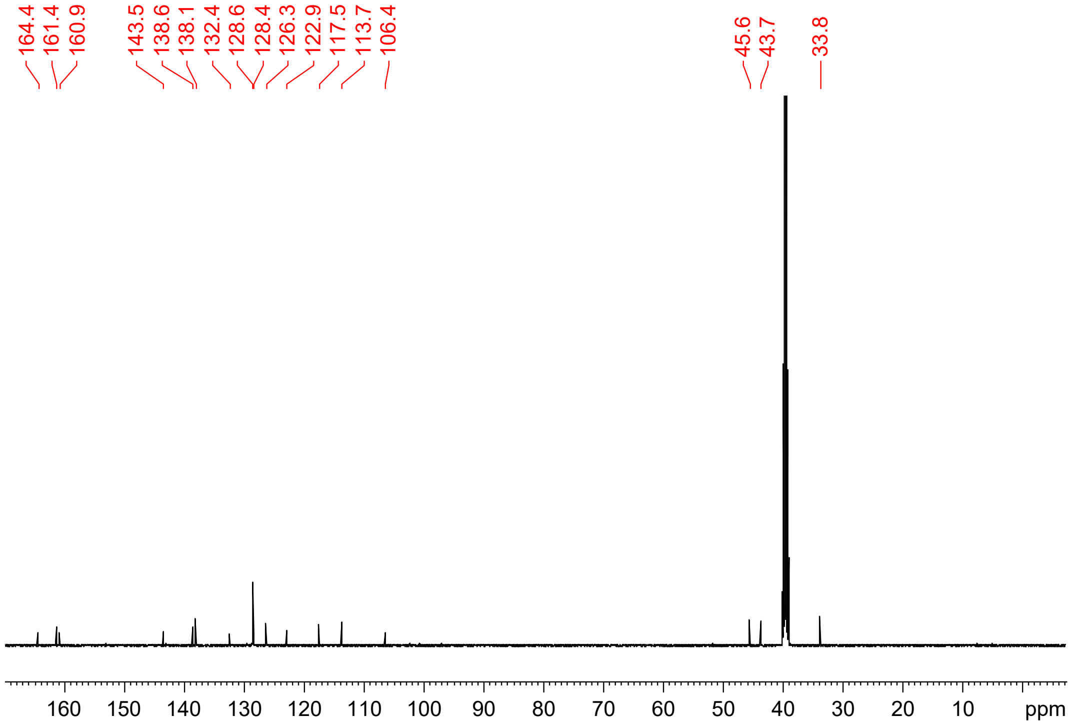

Figure S38. ^{13}C NMR spectrum (125 MHz, DMSO-d_6) of **12**

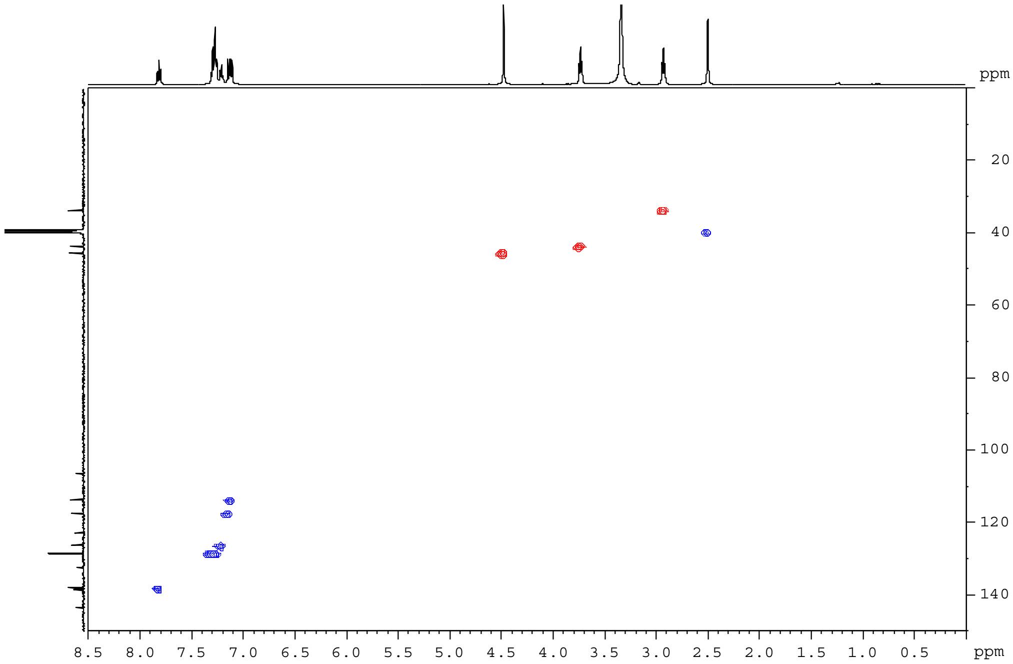

Figure S39. HSQC spectrum (500 MHz, DMSO-d_6) of **12**

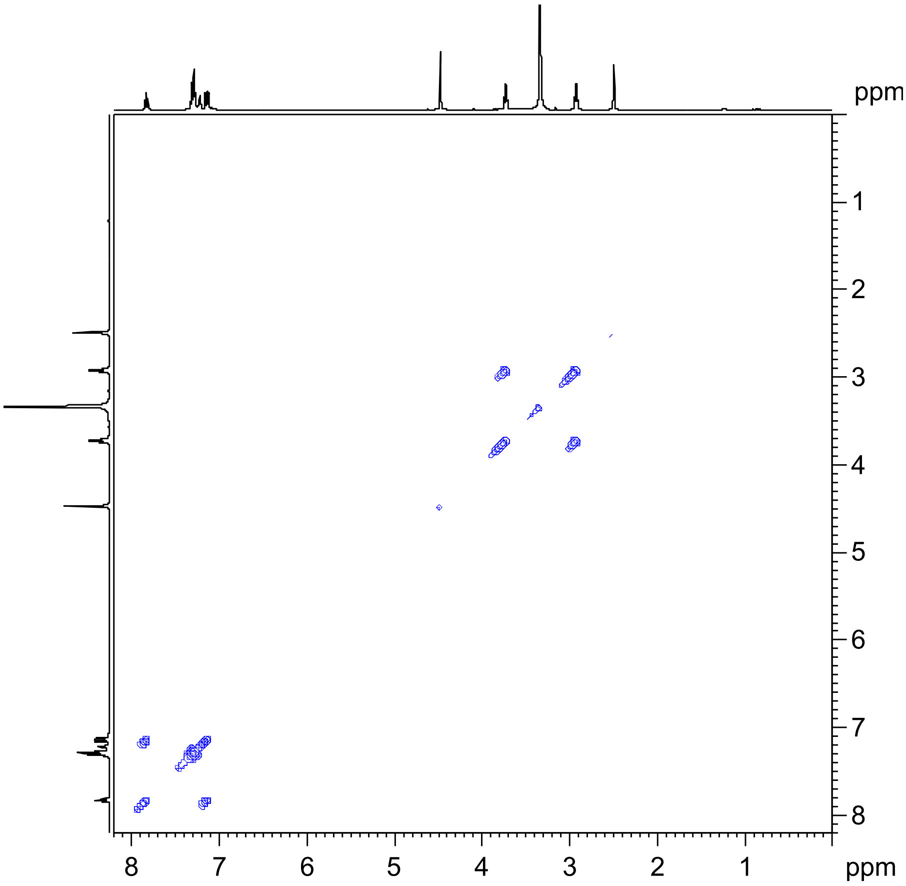

Figure S40. ^{1}H -^{1}H COSY spectrum (500 MHz, DMSO-d_6) of **12**

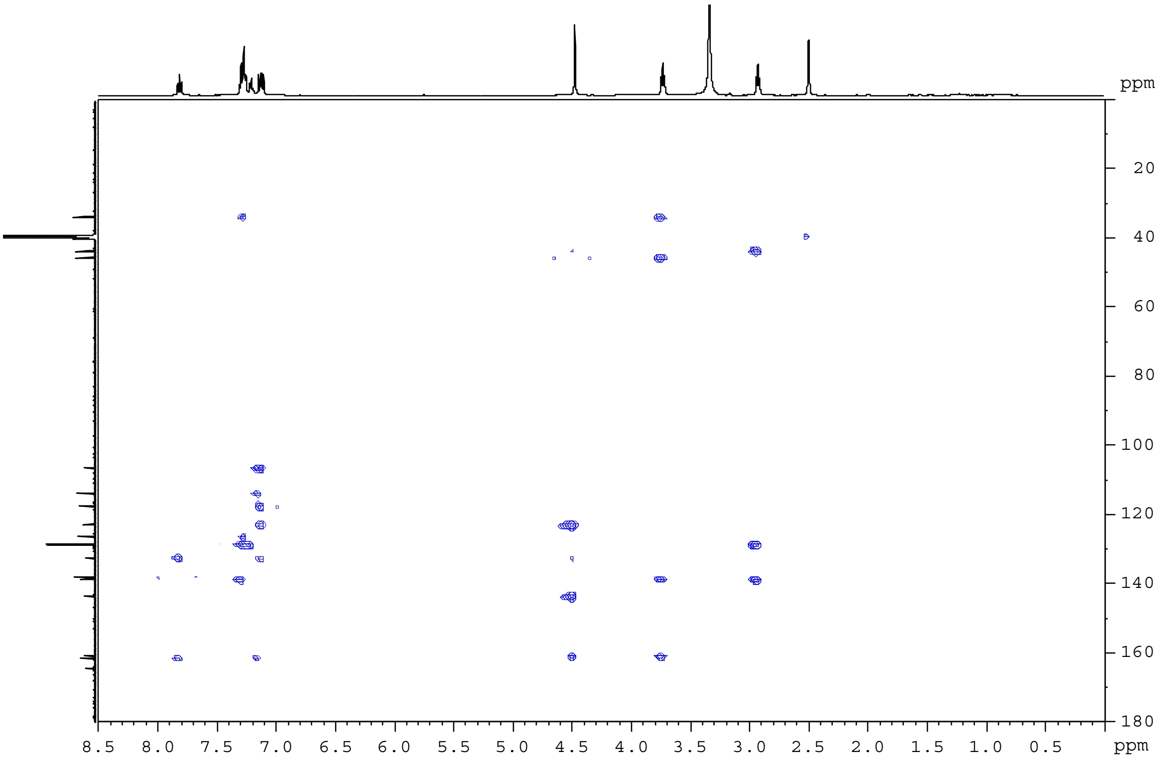

Figure S41. HMBC spectrum (500 MHz, DMSO-d_6) of **12**

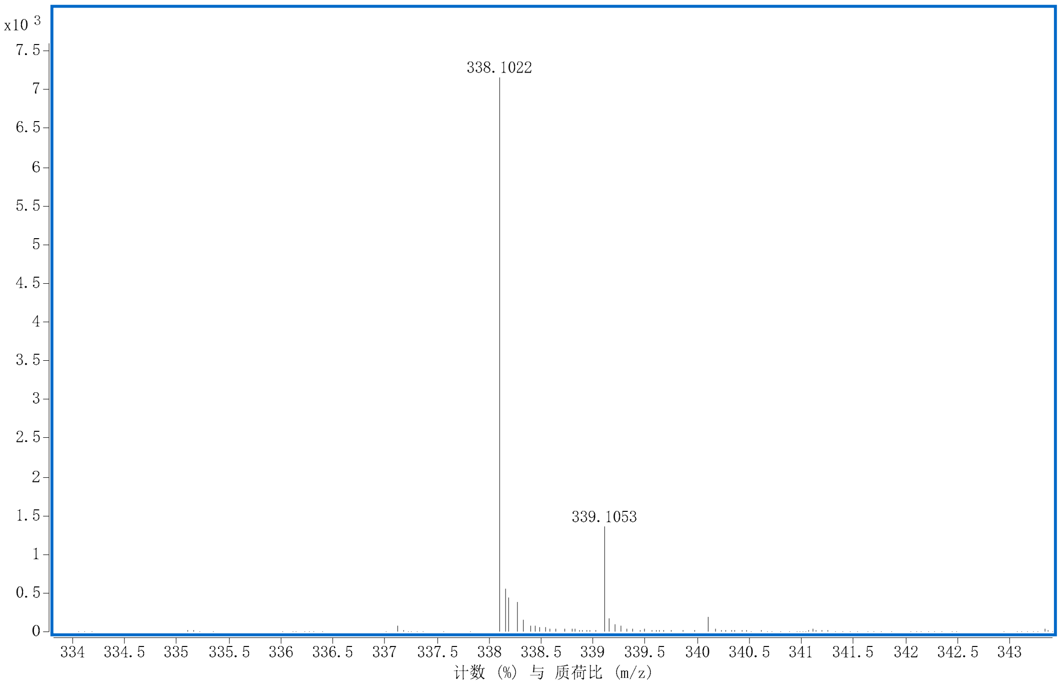

Figure S42. HRESIMS spectrum for **13**

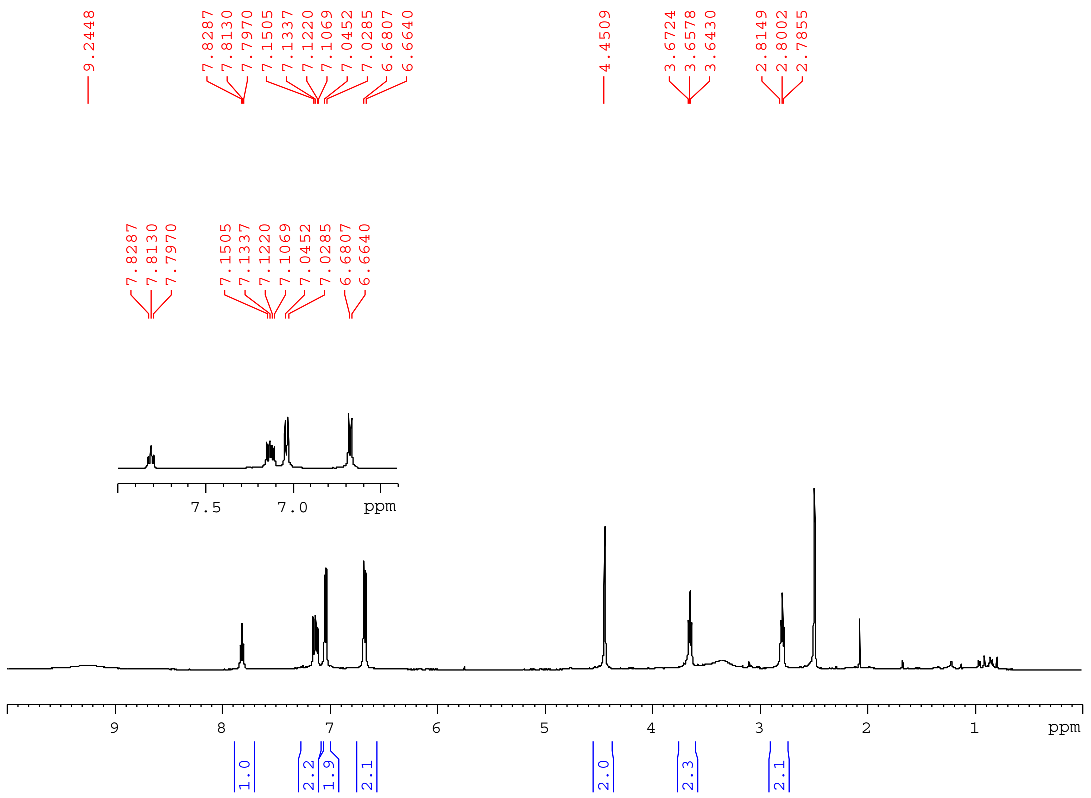

Figure S43. ^{1}H NMR spectrum (500 MHz, DMSO-d_6) of **13**

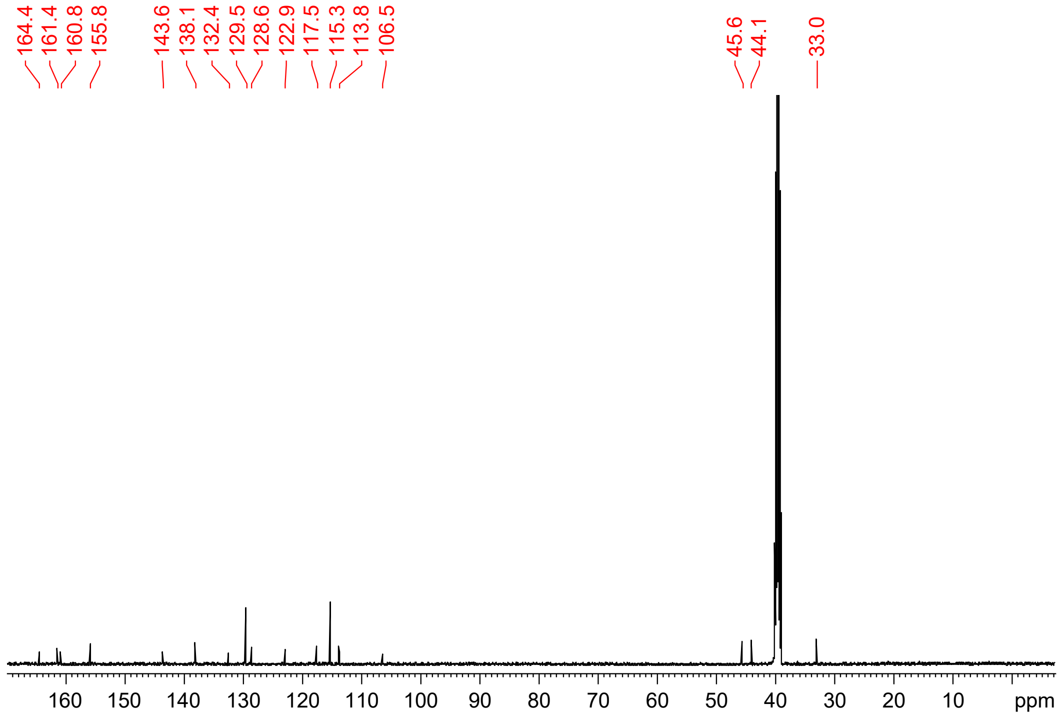

Figure S44. ^{13}C NMR spectrum (125 MHz, DMSO-d_6) of **13**

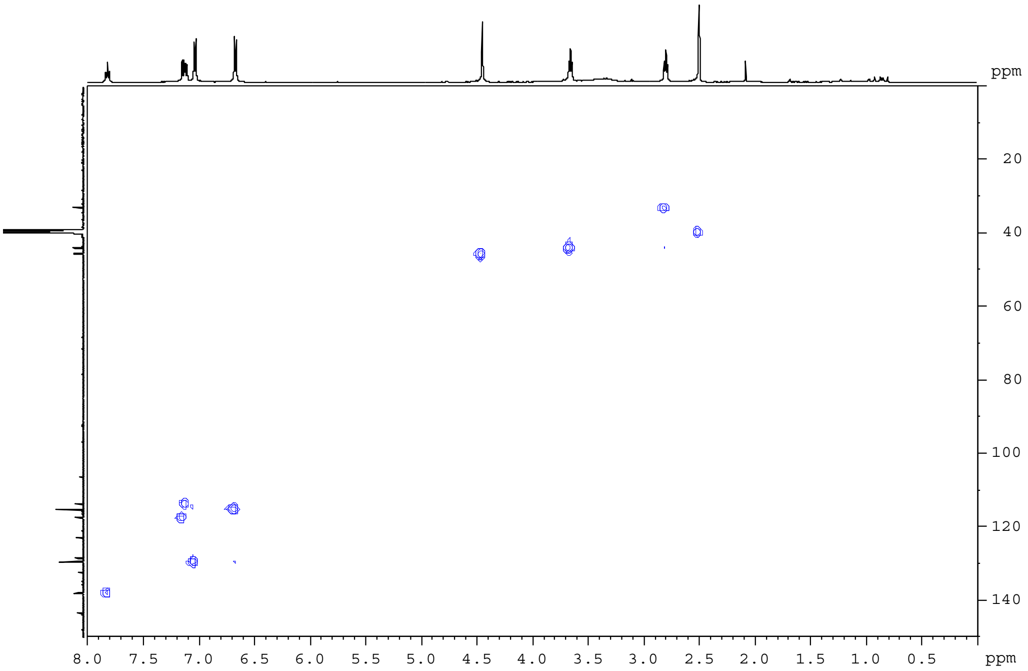

Figure S45. HSQC spectrum (500 MHz, DMSO-d_6) of **13**

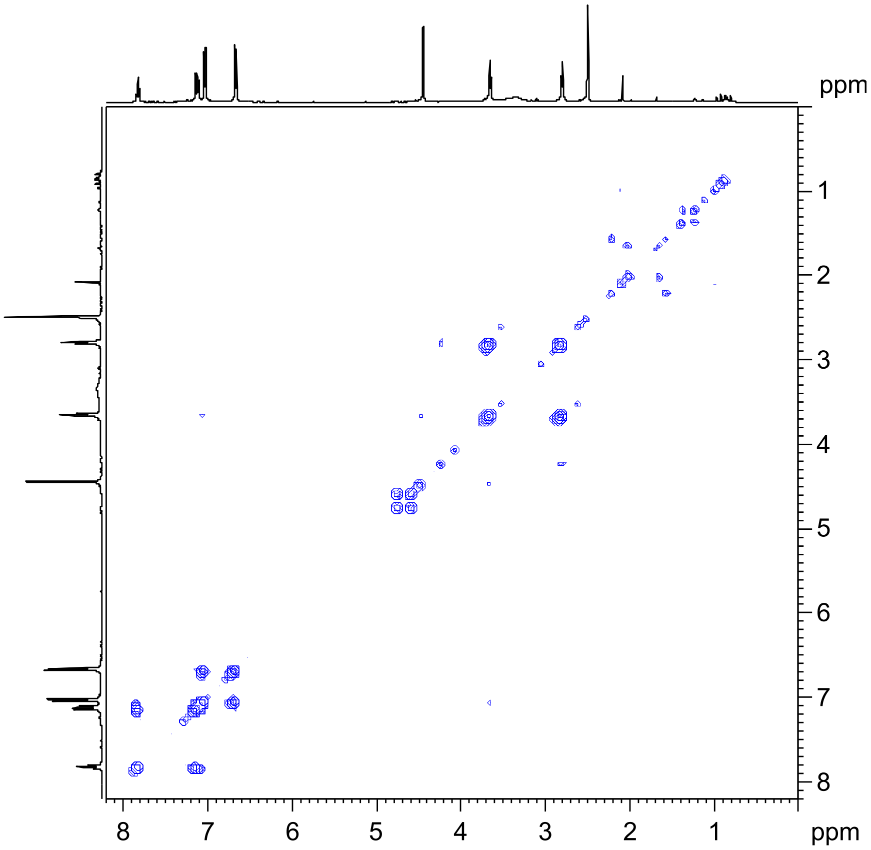

Figure S46. ^{1}H -^{1}H COSY spectrum (500 MHz, DMSO-d_6) of **13**

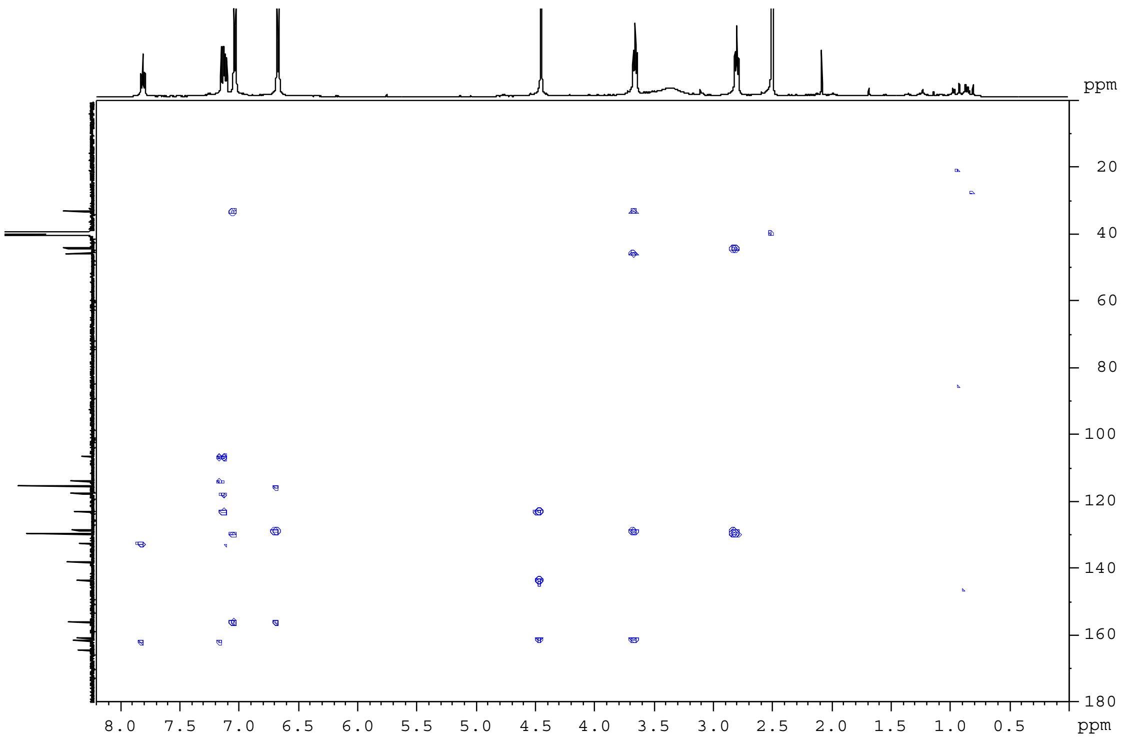

Figure S47. HMBC spectrum (500 MHz, DMSO-d_6) of **13**

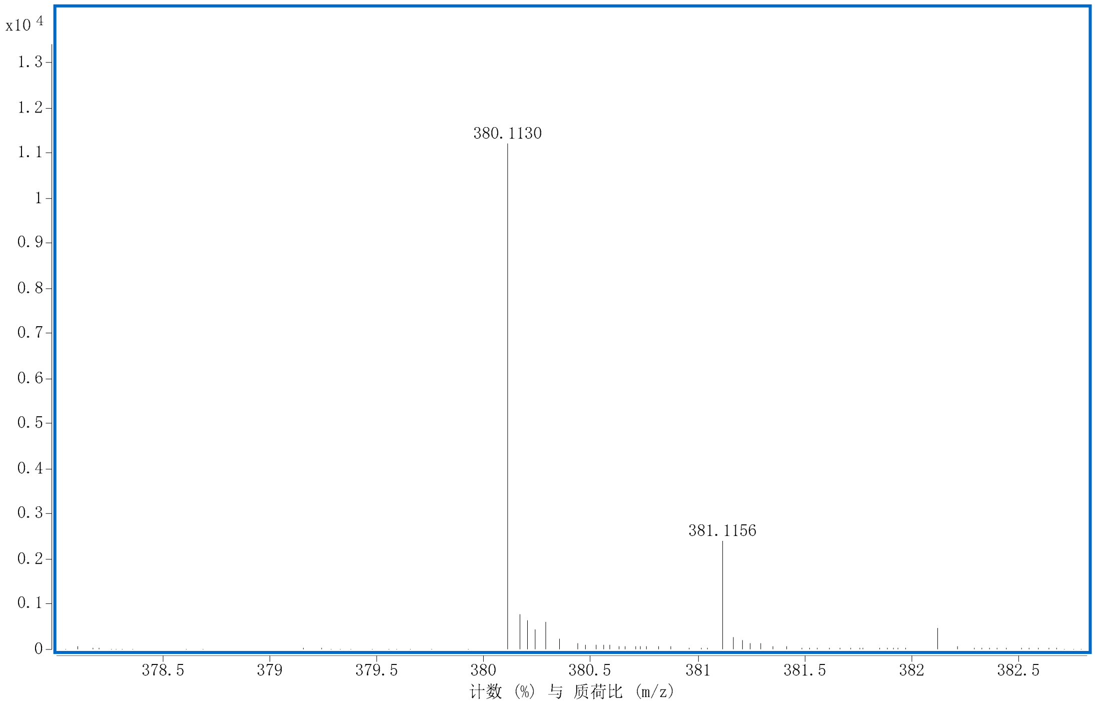

Figure S48. HRESIMS spectrum for **14**

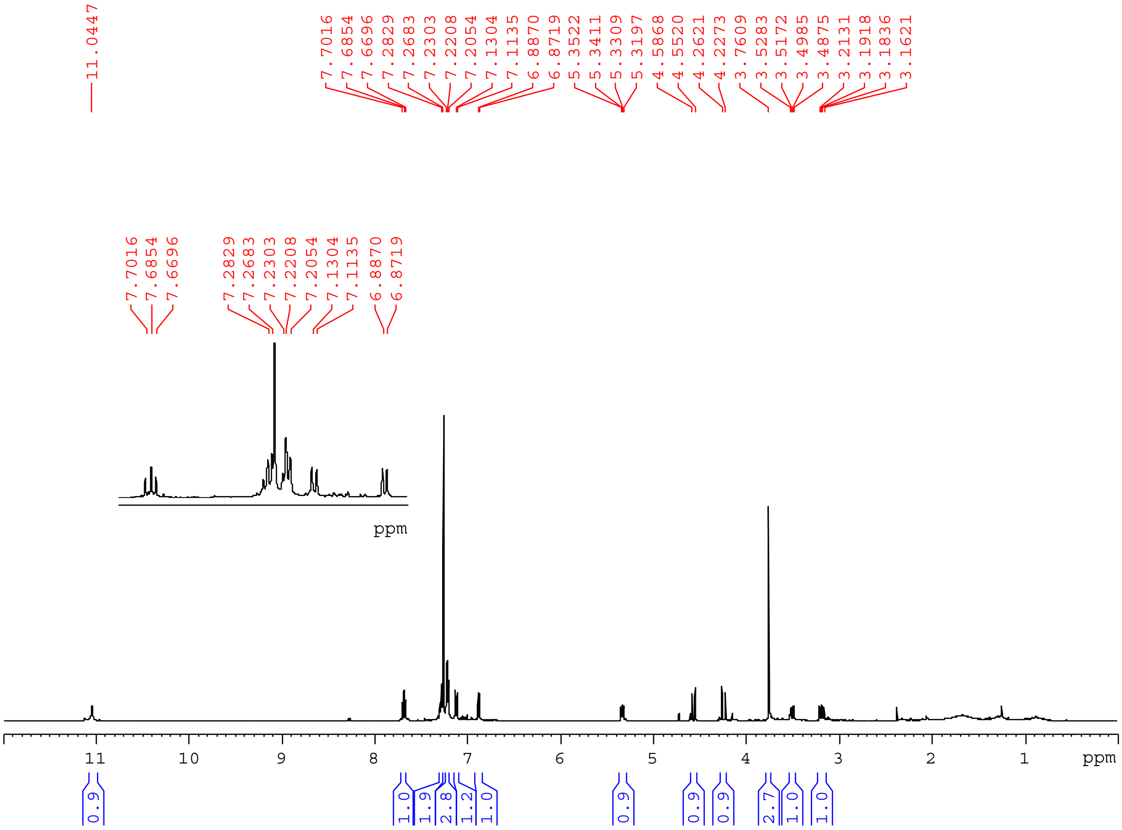

Figure S49. ^{1}H NMR spectrum (500 MHz, $CDCl_3$) of **14**

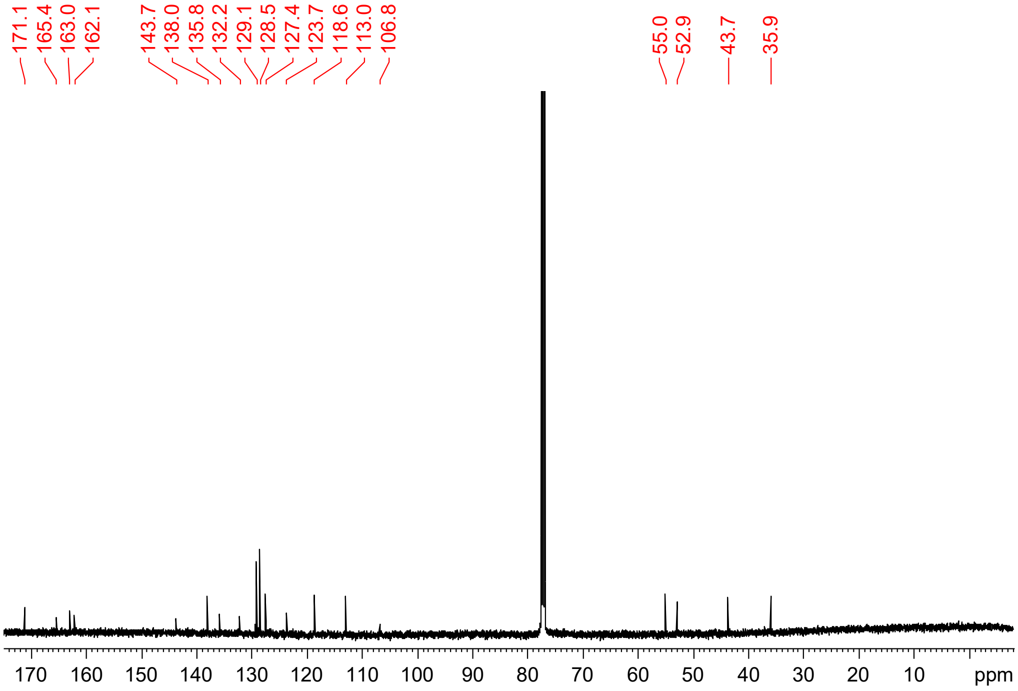

Figure S51. ^{13}C NMR spectrum (125 MHz, $CDCl_3$) of **14**

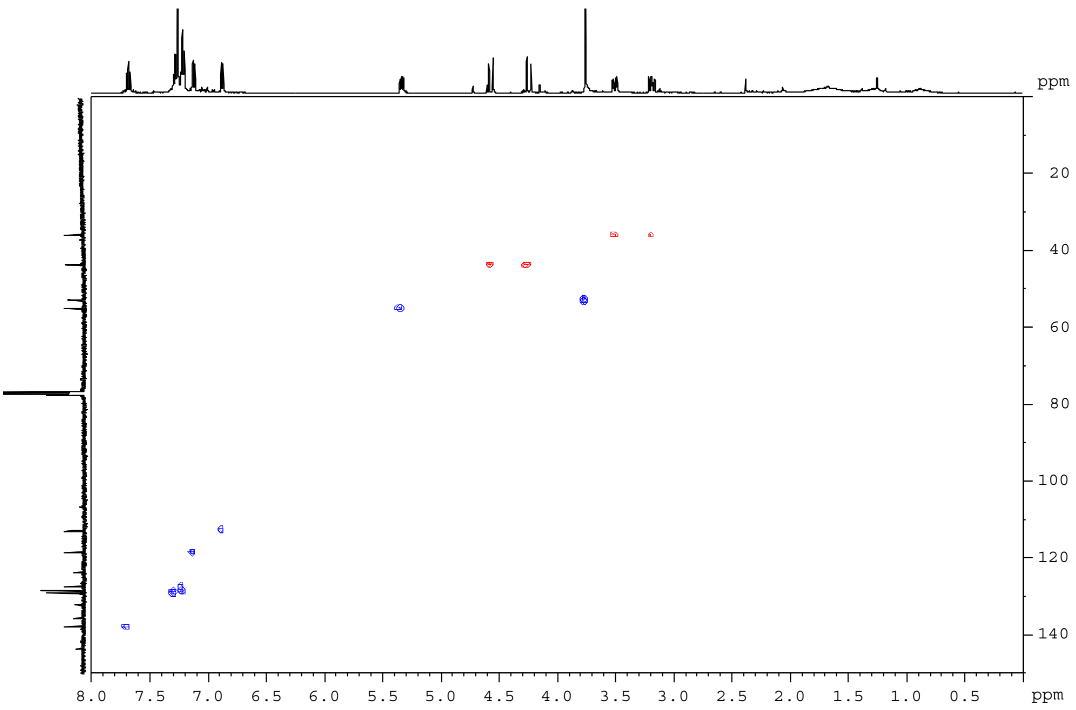

Figure S52. HSQC spectrum (500 MHz, $CDCl_3$) of **14**

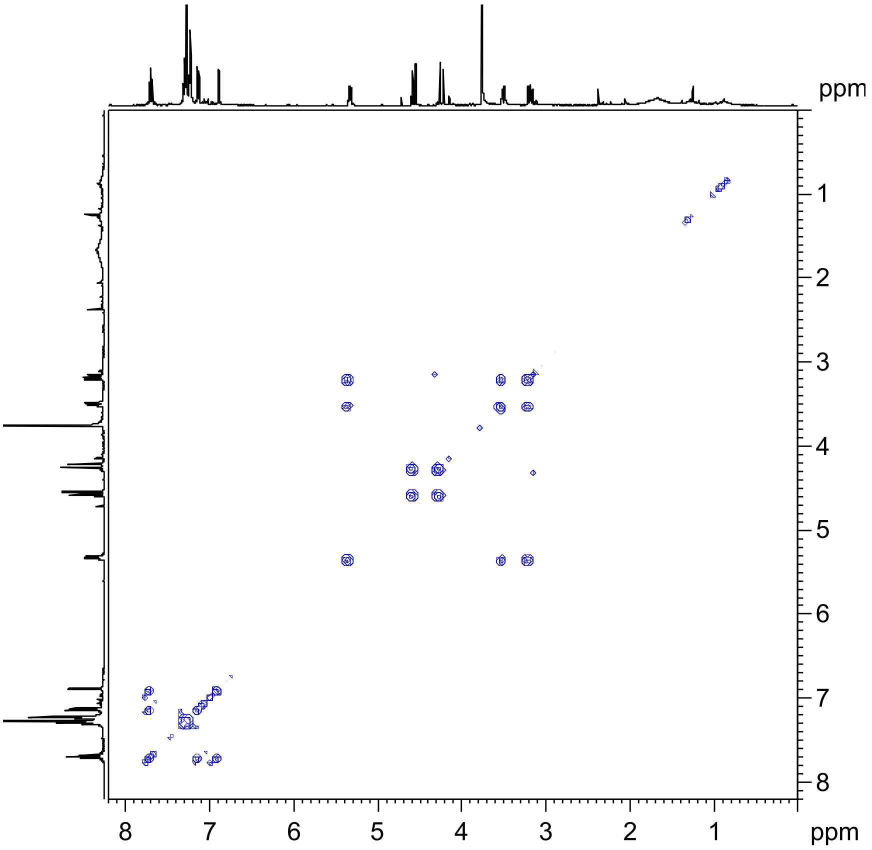

Figure S53. ^1H -^1H COSY spectrum (500 MHz, $CDCl_3$) of **14**

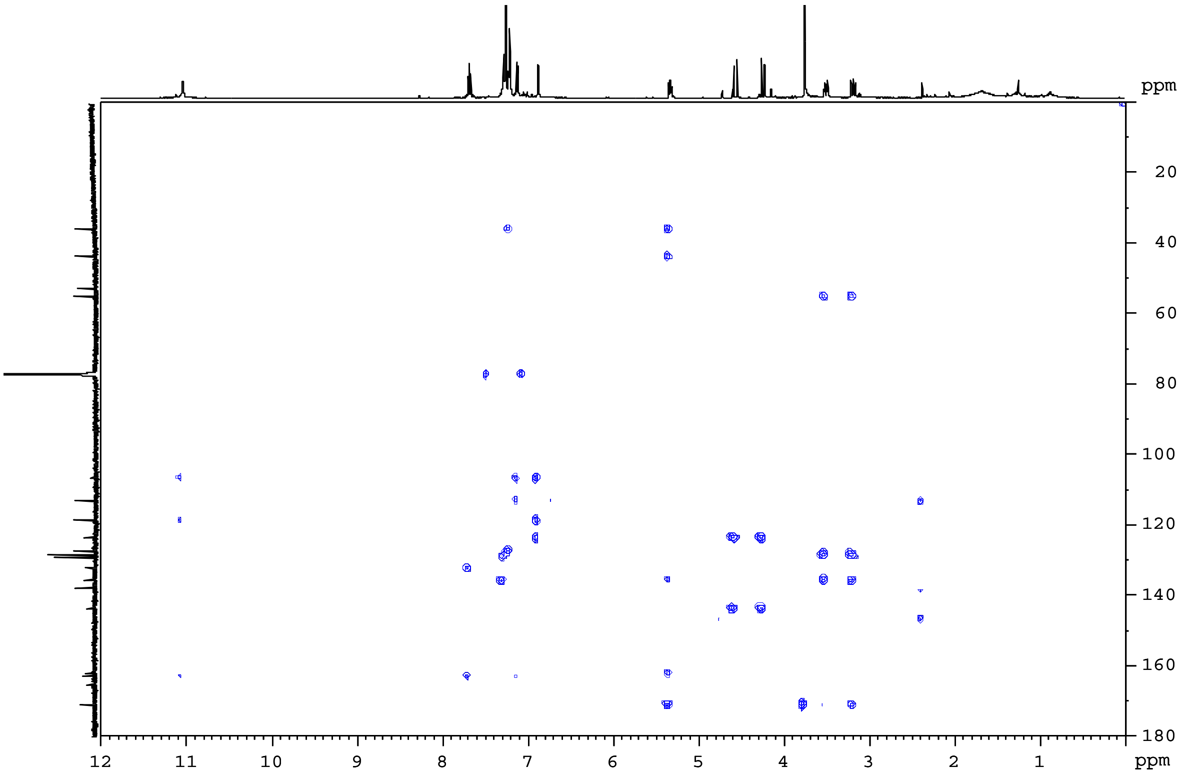

Figure S54. HMBC spectrum (500 MHz, $CDCl_3$) of **14**

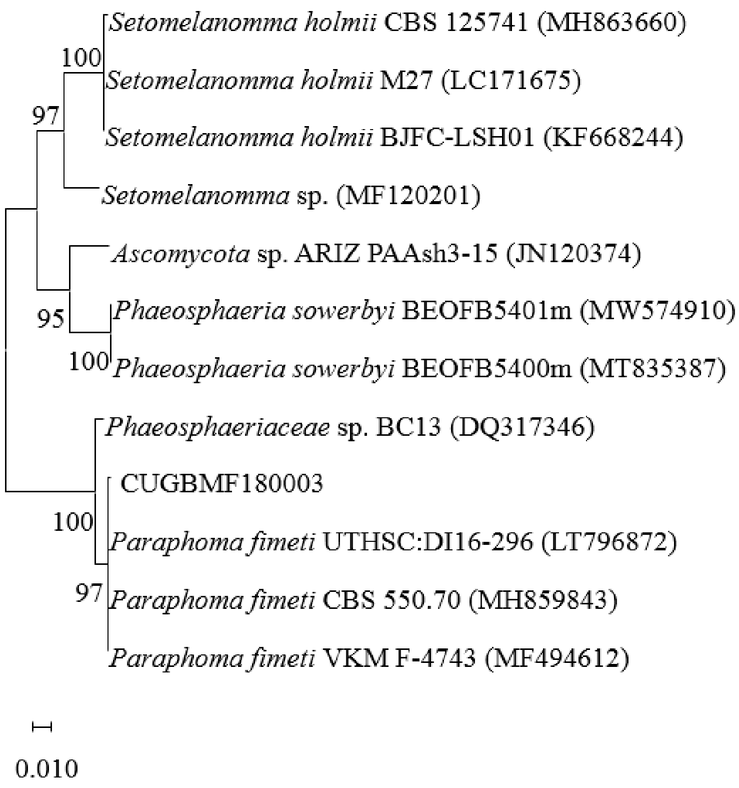

Figure S55. Neighbor-joining phylogenetic tree of CUGBMF180003 and its most related type strains based on internal transcribed spacer region (ITS) from NCBI ITS database. Numbers at nodes indicate levels of bootstrap support (%) based on a neighbor joining analysis of 1,000 resampled datasets; only values >50 % are given. NCBI accession numbers are given in parentheses. Bar 0.010 nucleotide substitutions per site.
